# *De novo* transcriptome sequencing in *Bixa orellana* to identify genes involved in methylerythritol phosphate, carotenoid and bixin biosynthesis

**DOI:** 10.1186/s12864-015-2065-4

**Published:** 2015-10-28

**Authors:** Yair Cárdenas-Conejo, Víctor Carballo-Uicab, Meric Lieberman, Margarita Aguilar-Espinosa, Luca Comai, Renata Rivera-Madrid

**Affiliations:** Centro de Investigación Científica de Yucatán, A. C. Calle 43 No. 130, Col. Chuburná de Hidalgo, 97200 Mérida, Yucatán Mexico; Plant Biology and Genome Center, University of California, Davis, CA 95616 USA

**Keywords:** Annatto, *Bixa orellana*, Lipstick tree, Transcriptome, Bixin synthesis, Carotenoids

## Abstract

**Background:**

Bixin or annatto is a commercially important natural orange-red pigment derived from lycopene that is produced and stored in seeds of *Bixa orellana* L. An enzymatic pathway for bixin biosynthesis was inferred from homology of putative proteins encoded by differentially expressed seed cDNAs. Some activities were later validated in a heterologous system. Nevertheless, much of the pathway remains to be clarified. For example, it is essential to identify the methylerythritol phosphate (MEP) and carotenoid pathways genes.

**Results:**

In order to investigate the MEP, carotenoid, and bixin pathways genes, total RNA from young leaves and two different developmental stages of seeds from *B. orellana* were used for the construction of indexed mRNA libraries, sequenced on the Illumina HiSeq 2500 platform and assembled de novo using Velvet, CLC Genomics Workbench and CAP3 software. A total of 52,549 contigs were obtained with average length of 1,924 bp. Two phylogenetic analyses of inferred proteins, in one case encoded by thirteen general, single-copy cDNAs, in the other from carotenoid and MEP cDNAs, indicated that *B. orellana* is closely related to sister Malvales species cacao and cotton. Using homology, we identified 7 and 14 core gene products from the MEP and carotenoid pathways, respectively. Surprisingly, previously defined bixin pathway cDNAs were not present in our transcriptome. Here we propose a new set of gene products involved in bixin pathway.

**Conclusion:**

The identification and qRT-PCR quantification of cDNAs involved in annatto production suggest a hypothetical model for bixin biosynthesis that involve coordinated activation of some MEP, carotenoid and bixin pathway genes. These findings provide a better understanding of the mechanisms regulating these pathways and will facilitate the genetic improvement of *B. orellana*.

**Electronic supplementary material:**

The online version of this article (doi:10.1186/s12864-015-2065-4) contains supplementary material, which is available to authorized users.

## Background

The nutritional and pharmaceutical potential of plant secondary metabolites is vast and still largely unexplored. Many plant species utilized for production of secondary metabolites that are important components of human diet, animal feed, medicines, biopesticides, and bioherbicides, have been subject of limited research and genetic improvement. This is the case of *Bixa orellana* L., achiote in Mexico, a species belonging to the Bixaceae family within the order Malvales [1, 2]. *Bixa orellana* is a tropical perennial and ligneous plant of great agroindustrial interest due to its high content of bixin, an apocarotenoid located mainly in the seeds. Bixin or annatto is an orange-red pigment that has been used for many years as a dye in foods, such as dairy and bakery products, vegetable oils, and drinks [3]. The world demand for annatto is increasing together with the interest in natural food dyes.

Carotenoids are yellow to red pigments synthesized by microorganisms and plants. In plants, they accumulate in the plastids (chromoplasts) of flowers and fruits. These compounds have antioxidant functions in all organisms, including animals and fungi, and play an important role in protecting cells from damage of radicals such as singlet oxygen [4]. Carotenoids are the major source of vitamin A (retinol) in animals, and abscisic acid (ABA) in plants [5]. All carotenoids are synthesized by consecutive condensations of isopentenyl diphosphate (IPP), which in turn is synthesized through the plastidial methylerythritol phosphate (MEP) pathway [6, 7]. Seven enzymatic steps produce IPP from pyruvate and glyceraldehyde-3-phosphate [6, 7]. The first step in carotenoid biosynthesis is the head-to-head condensation of two geranylgeranyl diphosphate (GGDP) molecules to produce phytoene, catalyzed by phytoene synthase (PSY). Subsequently, four enzymes convert phytoene to lycopene via phytofluene, zeta-carotene and neurosporene: two desaturases introduce four double bonds (phytoene desaturase (PDS), and zeta-carotene desaturase (ZDS)), and two isomerases acting, respectively, on the 7/9-7′/9′ double bound (carotene cis-trans isomerase, CRTISO) and C15-15′ double bonds (ζ-carotene isomerase, Z-IZO) [8, 9]. The cyclization of lycopene denotes a central branch point in the carotenoid biosynthesis pathway, and the relative activity of epsilon-cyclase (ε-LYC) versus beta-cyclase (β-LYC) may determine the flow of carotenoids from lycopene to either α-carotene or β-carotene [8].

Apocarotenoids as bixin are derived from the oxidative cleavage of carotenoids, which might occur randomly through photo-oxidation or lipoxygenase co-oxidation [10]. At the same time, the enzymatic cleavage of carotenoids through specific carotenoid dioxygenases (CCDs) has also been proposed [10, 11]. Bixin is derived from the enzymatic cleavage of lycopene [12, 13]. A biosynthetic pathway for bixin has been proposed [12, 14] and supported using a heterologous expression system [12]. This identification, however, has not been supported by a full characterization. Three *B. orellana* cDNAs encoding the enzymes required for bixin synthesis derived from the linear C_40_ lycopene have been identified: lycopene cleavage dioxygenase (*BoLCD*), bixin aldehyde dehydrogenase (*BoBALDH*) and norbixin methyltransferase (*BonBMT*) [12].

In spite of the great economic importance of achiote, its transcriptome and the genes from MEP and carotenoid pathways remained uncharacterized. Before this work, we had only access to partial sequences of some genes [14, 15] obtained from expressed sequences tags (ESTs) isolated from a subtracted cDNA library made with RNA from immature seed and leaves [14]. The library identified clusters of transcripts corresponding to five genes of the MEP pathway: (1-Deoxy-D-xylulose-5-phosphate synthase (*DXS*), 1-Deoxy-D-xylulose-5-phosphate reductoisomerase (*DXR*), 2-C-Methyl-D-erythritol 4-phosphate cytidyltransferase (*MCT*), 4-Hydroxy-3-methylbut-2-en-1-yl diphosphate synthase (*HDS*)), the intermediate gene geranylgeranyl diphosphate synthase (*GGDS*), three genes of the carotenoid pathway (*PSY*, *PDS*, *ZDS*) and three genes of the bixin pathway (Carotene deoxygenase, aldehyde dehydrogenase and methyl transferase), which were overexpressed in immature seeds compared to leaves [14]. The limited genetic and molecular data available for *B. orellana*, is attributable in part to its high amounts of polyphenols, pigments and gummy polysaccharides, which complicate nucleic acid purification. To overcome this difficulty, Rodríguez-Ávila and co-workers developed a protocol to isolate total RNA from multiple tissues of *B. orellana* [16] that proved effective for single gene assay expression analysis. Here we leverage it together with high throughput sequencing, to assemble a transcriptome for this plant. We demonstrate its use to identify the MEP, carotenoid and bixin pathway genes.

## Results

### *De novo* sequence assembly of *B. orellana* transcriptome

**Fig. 1:**
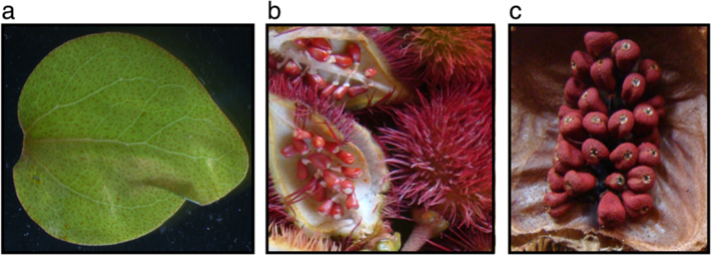
*Bixa orellana* tissues used as mRNA sources for sequencing and transcriptome assembly. **a** Leaf, (**b**) immature seed, and (**c**) mature seed

To investigate the MEP, carotenoid, and bixin pathways genes, we sequenced the transcriptome of *B. orellana* using mRNA from young leaves and two different developmental stages of seeds (immature and mature) (Fig. 1). From the isolated mRNA we constructed indexed cDNA libraries and sequenced them on the Illumina HiSeq 2500 platform. The reads were assembled de novo using Velvet [17], CLC Genomics Workbench (http://www.clcbio.com) and CAP3 [18] software. In a strategy similar to that of Ashrafi et al., [19], separate Velvet and CLC assemblies were carried out, followed by merging the resulting contigs through CAP3. This strategy optimized the number of different cDNAs assembled, their overall length and the length of the encoded open reading frames (ORF). The final CAP3 set consisted of 52,549 contigs with an N50 of 2,294 bp. The average length of the contigs was 1,924 bp, ranging from 301 to 25,617 bp (Table 1). The contig size distribution showed that 41,209 contigs (78.4 %) were larger than 1,000 bp, 65 contigs (0.1 %) had a greater length than 10,000 bp and 11,275 contigs (21.5 %) were shorter than 1,000 bp. Using orf_finder software from WebMGA server [20], we performed an ORF search in order to determine the approximate number and size of the proteins coded in the transcriptome. A total of 25,555 ORFs ≥ 300 b were detected, the average length was 1,578.5 b and the longest had 11,322 b (Table 1).

**Table 1:** Assembly statistics

Total number of contigs	52,549
Transcriptome size(nt)	101,106,695
Longest contig	25617
Shortest contig	301
Average contigs length(nt)	1,924
N50(nt)	2,294
Total number of ORF	25,555
Average ORF length(nt)	1578.5
Longest ORF(nt)	11,322
Shortest ORF(nt)	300

The assemblathon_stats perl scripts version 2 and ORF_finder were used to compute assembly statistics

### Evolutionary relationship of *Bixa orellana*

**Fig. 2:**
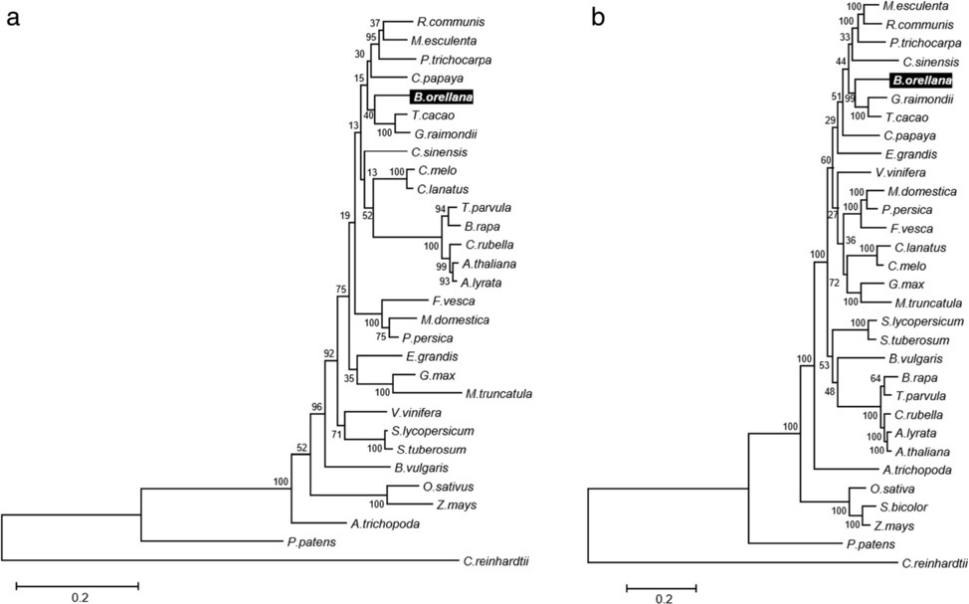
Evolutionary relationship of *B. orellana.* **a** Phylogenetic analysis based on alignment of concatenated proteins encoded by sets of 13 single copy genes [19] from 28 plant species and one moss species. **b** Phylogenetic analysis based on alignment of concatenated enzymes of the carotenoid/MEP pathways in 29 plant species and one moss species. Numbers near the branch points represent the bootstrap value produced by 1000 replications. The trees are drawn to scale, with branch lengths proportional to the number of substitutions per site. Single-celled green alga *Chlamydomonas reinhardtii* was used as an outgroup. Protein sequences and plant species used are listed in Additional file 1: Table S9

In order to elucidate the evolutionary relationship of *B. orellana*, a phylogenetic analysis of 13 proteins encoded by presumed single-copy genes in most plants, identified by Duarte and co-workers [21], was carried out. These single-copy genes yielded well-resolved tree topologies [21, 22]. The phylogenetic analysis grouped achiote in the Malvidae clade, in close relationship with cotton (*Gossypium raimondii*) and cacao (*Theobroma. cacao*) (Fig. 2a).

### Blast search in public databases

**Fig. 3:**
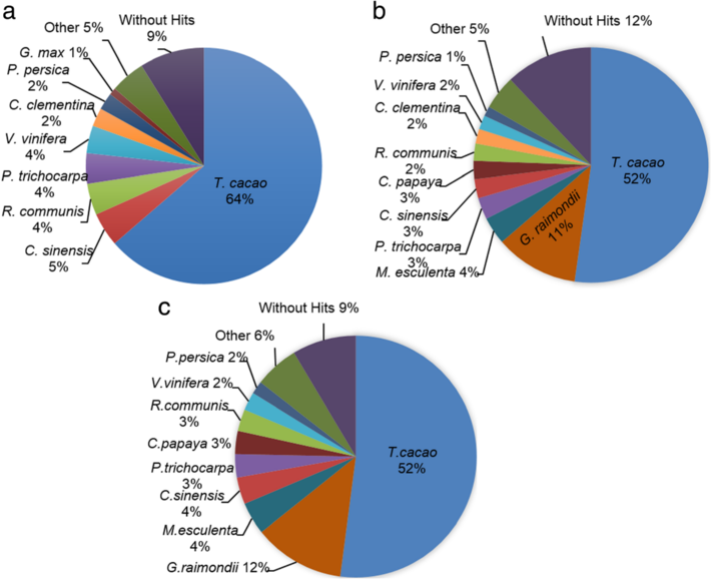
BLASTX top-hits species distribution. The *B. orellana* transcriptome was compared to: (**a**) the NCBI RefSeq plant protein database, (**b**) the Phytozome protein database version 10, and (**c**) the PLAZA protein database version 3.0. The percent of contigs producing hit for each species is marked after the species scientific name

We compared achiote transcriptome (52,549 contigs) to three protein databases, NCBI Plant Protein Reference sequence (RefSeq), Phytozome, and PLAZA 3.0, using the BLASTX algorithm with a cutoff e-value of 1e-6. The search against RefSeq exhibited a total of 47,894 contigs (91 %) with significant hits, while comparisons against the Phytozome and PLAZA 3.0 databases showed that 46,232 contigs (88 %) and 48,047 contigs (91 %) had significant hits, respectively. BLAST hits from the RefSeq comparison were distributed between 28 plant species. Eight plant species had ≥ 1 % transcriptome contigs hits (Fig. 3a). Hits obtained by the Phytozome comparison were distributed between 35 plant species; ten of them had ≥ 1 % transcriptome contigs blast hit (Fig. 3b). Twentyeight plant species were represented in the 48,045 BLAST hits obtained by PLAZA 3.0 comparison, and 9 out of the 28 had ≥ 1 % transcriptome contigs blast hit (Fig. 3c). In all comparisons, cacao (*T. cacao*) provided the best BLAST hits: 33,442 contigs (64 %) when the transcriptome was compared with the RefSeq database, 27,454 contigs (52 %) compared with the Phytozome database and 27,362 contigs (52 %) with the PLAZA 3.0 database (Fig. 3). The second best represented plant species in the BLAST results was orange (*Citrus sinensis*) with 2446 contigs from the RefSeq comparison and cotton (*G. raimondii*) with 6016 and 6410 contigs displayed by Phytozome and PLAZA 3.0 comparisons, respectively (Fig. 3). BLASTX results for transcriptome comparisons are available in Additional file 1: Table S1.

To compare the achiote transcriptome with a previous achiote EST library created by Jako and co-workers [14], we performed a bidirectional BLASTN. Jako and co-workers library has 954 sequences registered, the longest sequence is 691 bp and the shortest is 50 bp with a mean sequence size of 355 bp [14]. Using the EST library as a query, we found that 714 EST sequences (74.8 %) had BLAST hits, with an average identity of 99 % and identity range between 90.91 and 100 % (Additional file 1: Table S2). Whereas, 583 contigs (1.1 % of transcriptome) had hits to the EST library, with a high average identity of 98.6 %. The identity range was between 82.77 % and 100 % (Additional file 1: Table S2).

### Functional annotation of gene ontology

**Fig. 4:**
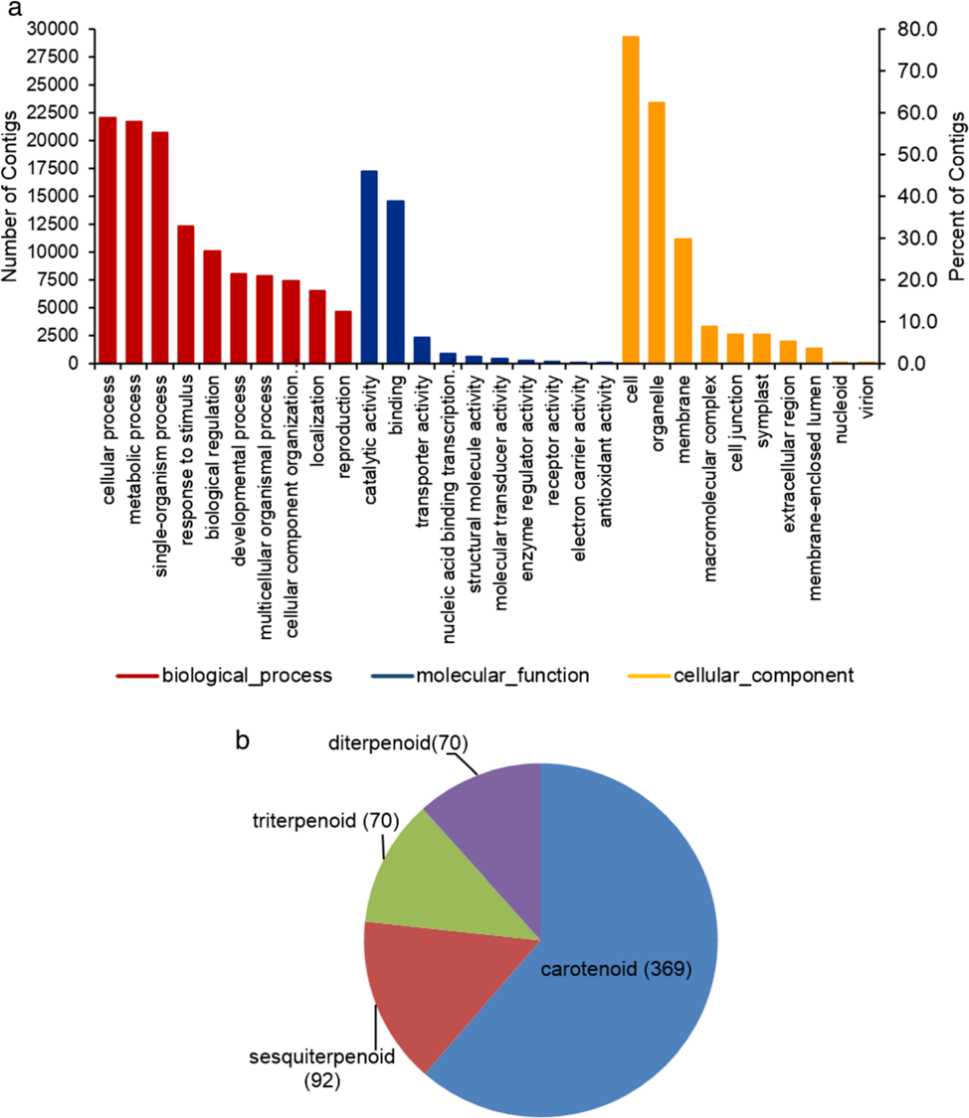
Gene ontology (GO) annotation. **a** The top ten GO descriptions in the three main categories, biological process, cellular component and molecular function. **b** Contig distribution for terpenoid metabolic process (GO:0006721). Number of contigs per description are in brackets

We used the BLASTX results of the achiote transcriptome against the RefSeq database to extract Gene Ontology (GO) terms with Blast2GO software. 38,076 contigs (80 %) with significant hits out of 47,894 were annotated and classified in 7461 GO terms. These GO terms were split in the three main GO categories, “Biological process” (4314 Go terms), “Molecular function” (2485 terms) and “Cellular component” (665 GO terms). In “biological process”, the top three GO descriptions from level 2 were “cellular process” with 22,066 contigs, “metabolic process” with 21,664 contigs and “single-organism process” with 20,762. In “molecular function”, the largest description was “catalytic activity” with 17,260 contigs followed by “binding” and “transporter activity”. In reference to the “cellular component” term, the most represented descriptions were “cell”, “organelle” and “membrane” with 29,327, 23,421 and 11,200 contigs, respectively (Fig. 4a).

With regard to carotenoids biosynthesis, 601 contigs from 38,076 with GO annotation were classified in “terpenoid metabolic process” (GO:0006721, Fig. 4b). 369 contigs (61.4 %) from this description belong to GO term “carotenoid” (GO:0016117). The rest of 232 contigs included in GO:0006721 were split in three descriptions, “diterpenoid”, “triterpenoid”, and “sesquiterpenoid”. GO annotation is available in Additional file 1: Table S3.

### KEGG pathway annotation

**Fig. 5:**
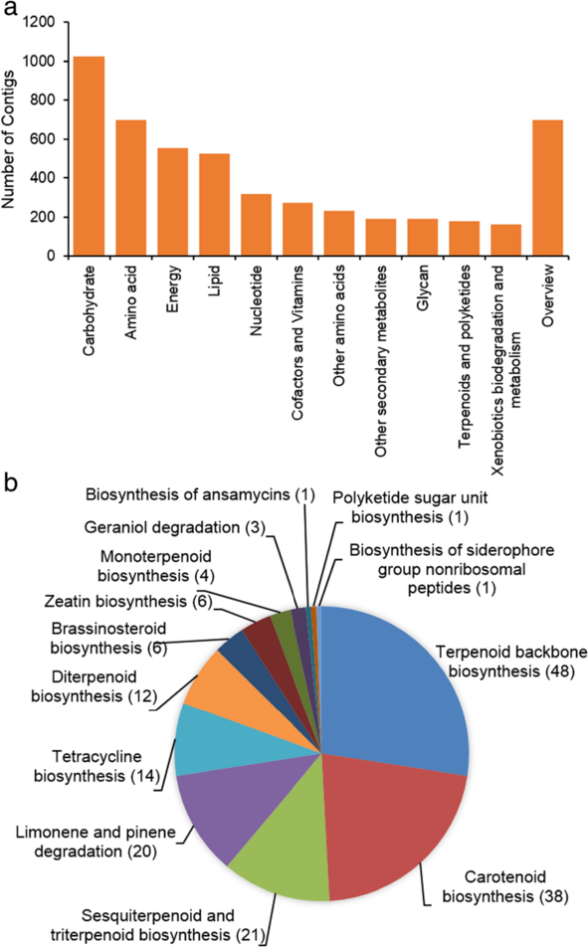
Kyoto Encyclopedia of Genes and Genomes (KEGG) annotation. **a** Classification based on metabolism categories. **b** Classification based on metabolism of terpenoids and polyketides. Number of contigs per pathway is in brackets

In order to assign biochemical pathways to *B. orellana* transcriptome, a functional pathway annotation was performed against the Kyoto Encyclopedia of Genes and Genomes (KEGG). The KEGG annotation was carried out with the KAAS server (KEGG Automatic Annotation Server) by BLAST comparisons against the KEGG GENES database. When the file with 52,549 contigs of transcriptome was uploaded to the server, 8698 were assigned to 3092 enzymes. The five main KEGG biochemical pathways were represented: metabolism (2349 contigs), genetic information processing (2082), organism system (851), cellular processes (764) and environmental information processing (783). In metabolism pathways, 2349 contigs were distributed in 5058 hits (Fig. 5a). The top three groups of metabolism pathways were “carbohydrate metabolism” with 1021 hits against 190 enzymes, followed by “amino acid metabolism” with 700 hits in 183 enzymes. The third group called “overview”, which included Carbon metabolism, 2-Oxocarboxylic acid metabolism, Fatty acid metabolism, Biosynthesis of amino acids and Degradation of aromatic compounds), had 506 hits and 175 enzymes.

In the terpenoids and polyketides pathways, which include the carotenoid pathways, 175 contigs could be associated with 75 enzymes (Fig. 5b). The largest pathway with 48 contigs was “Terpenoid backbone biosynthesis”, which includes enzymes from the MEP and mevalonate pathways. The carotenoid pathway was the second most represented group with 38 contigs and 17 enzymes. The twelve enzymes belonging to the carotenoid pathway were: PSY, PDS, 15-Z-ISO, ZDS, CRTISO, β-LYC, ε-LYC, β-carotene hydroxylase (βCH), cytochrome P450-type monooxygenase 97A (CYP97A3), cytochrome P450-type monooxygenase 97C1 (CYP97C1), zeaxanthin epoxidase (ZEP) and violaxanthin de-epoxidase (VDE). The five remaining enzymes are associated to derivate compounds of carotenes: capsanthin/capsorubin synthase (CCS1), 9-cis-epoxycarotenoid dioxygenase (NCED), xanthoxin dehydrogenase (ABA2), abscisic-aldehyde oxidase (AAO3) and abscisic acid 8′-hydroxylase. KEGG annotation is available in Additional file 1: Table S4.

### Identification of MEP and carotenoid pathways cDNAs from *B. orellana* transcriptome

To identify and isolate the cDNAs encoding proteins of the MEP and carotenoid pathway, a Local TBLASTN search against the achiote transcriptome was performed using homologous proteins from *Arabidopsis thaliana*, *G. raimondii* and *T. cacao* followed by a phylogenetic analysis of each putative protein. The search allowed us to identify the cDNAs encoding the seven canonical enzymes in the MEP pathway, the cDNAs encoding the 14 core enzymes of the carotenoid pathways and the cDNAs encoding intermediate pathway proteins isopentenyl diphosphate isomerase (*BoIDI*) and *BoGGDS* (Table 2).

**Table 2:** Identified cDNA from MEP, carotenoid and bixin pathways

Description	Jako Hits	GenBank Accession no.
BoDXS1 (1-Deoxy-D-xylulose-5-phosphate synthase)	0	KT358983
BoDXS2a	2	KT358984
BoDXS2b	0	KT358985
BoDXS3	0	KT358986
BoDXR (1-Deoxy-D-xylulose-5-phosphate reductoisomerase)	2	KT358987
BoMCT (2-C-Methyl-D-erythritol 4-phosphate cytidyltransferase)	0	KT358988
BoCMK ( 4-Diphosphocytidyl-2-C-methyl-D-erythritol kinase)	1	KT358989
BoMDS (2-C-Methyl-D-erythritol 2,4-cyclodiphosphate synthase)	0	KT358990
BoHDS 4-Hydroxy-3-methylbut-2-en-1-yl diphosphate synthase)	3	KT358991
BoHDR (4-Hydroxy-3-methylbut-2-enyl diphosphate reductase)	1	KT358992
BoIDI (Isopentenyl diphosphate isomerase)	0	KT358993
BoGGDS (Geranylgeranyl diphosphate synthase)	4	KT358994
BoPSY1 (Phytoene synthase)	1	KT358995
BoPSY2	1	KT358996
BoPDS1 (Phytoene desaturase)	9	KT358997
BoPDS2^a^	0	KT358998
BoZ-ISO (15-cis-ζ-carotene isomerase)	0	KT358999
BoZDS (ζ-carotene desaturase)	8	KT359000
BoCRTISO1a (Carotene cis-trans isomerase)	0	KT359001
BoCRTISO1b	0	KT359002
BoCRTIOS2	0	KT359003
Boβ-LYC1 (Lycopene β-cyclase)	0	KT359004
Boβ-LYC2	0	KT359005
Boε-LYC^a^ (Lycopene ε-cyclase)	0	KT359006
BoβCH1 (β-carotene hydroxylase)	0	KT359007
BoCYP97A3 (Cytochrome P450-type monooxygenase 97A3)	0	KT359008
BoCYP97C1 (Cytochrome P450-type monooxygenase 97C1)	0	KT359009
BoCYP97B3^a^ (Cytochrome P450-type monooxygenase 97B3)	0	KT359010
BoZEP1 (Zeaxanthin epoxidase)	0	KT359011
BoZEP2	0	KT359013
BoVDE1 (Violaxanthin de-epoxidase)	0	KT359014
BoVDE2^a^	0	KT359015
BoNSY (Neoxanthin synthase)	0	KT359016
BoCCD1-1 (Carotene cleavage dioxygenase 1-Copy1)	0	KT359018
BoCCD1-2	0	KT359019
BoCCD1-3	0	KT359020
BoCCD1-4^a^	0	KT359021
BoCCD4-1 (Carotene cleavage dioxygenase 4-Copy1)	0	KT359022
BoCCD4-2	9	KT359023
BoCCD4-3	16	KT359024
BoCCD4-4	0	KT359025
BoCCD4-5^a^	0	KT359026
BoALDH2B4 (aldehyde dehydrogenase 2B4)	0	KT359027
BoALDH2B7-1	0	KT359028
BoALDH2B7-2	0	KT359029
BoALDH2C4^a^	0	KT359030
BoALDH3F1	0	KT359031
BoALDH3F2	0	KT359032
BoALDH3H1-1	10	KT359033
BoALDH3H1-2	0	KT359035
BoALDH3I1	2	KT359036
BoALDH5F1	0	KT359038
BoALDH6B2-1	0	KT359039
BoALDH6B2-2	0	KT359040
BoALDH6B3	0	KT359041
BoALDH7B4	1	KT359042
BoALDH10A8	0	KT359043
BoALDH11A3	0	KT359044
BoALDH12A1	0	KT359045
BoALDH18B1-1	0	KT359046
BoALDH18B1-2	0	KT359047
BoALDH22A1	0	KT359048
BoSABATH1 (SABATH family Methyltransferase1)	0	KT359049
BoSABATH2	0	KT359050
BoSABATH3	3	KT359051
BoSABATH4	6	KT359052
BoSABATH5	0	KT359053
BoSABATH6	0	KT359054
BoSABATH7	0	KT359055
BoSABATH8	0	KT359056
BoSABATH9	0	KT359057
BoSABATH10^a^	0	KT359058
BoSABATH11	0	KT359059
BoSABATH12	0	KT359060

^a^Partial sequence

cDNAs encoding putative BoDXS in the MEP pathway were consistent with four genes: *BoDXS1, BoDXS3* and two paralogous copies of BoDXS2 (*BoDXS2a* and *BoDXS2b*). We identified cDNA consistent with single copy genes for the remaining MEP pathway enzymes: *BoDXR*, *BoMCT*, 4-Diphosphocytidyl-2-C-methyl-D-erythritol kinase (*BoCMK*), 2-C-Methyl-D-erythritol 2,4-cyclodiphosphate synthase (*BoMDS*), *BoHDS*, and 4-Hydroxy-3-methylbut-2-enyl diphosphate reductase (*BoHDR*). Also single copies were identified for the intermediate genes *BoIDI* and *BoGGPS*. Comparison to MEP pathways cDNAs isolated in the previous EST library [14] showed that *BoDXS2a*, *BoDXR1*, *BoCMK*, *BoHDS*, *BoHDR* and *BoGGDS* were common (Table 2).

In the carotenoid pathway, cDNAs characterization identified two gene copies for phytoene synthase (*BoPSY1* and *BoPSY2*), phytoene desaturase (*BoPDS1* and *BoPDS2*), β lycopene cyclase (*Boβ-LYC1* and *Boβ-LYC2*), zeaxanthin epoxidase (*BoZEP1* and *BoZEP2*) and violaxanthin de-epoxidase (*BoVDE1* and *BoVDE2*). The remaining carotenoid pathway genes were found in single copy, except *CRTISO* for which three copies were identified: *BoCRTISO2* and paralogous *BoCRTISO1a* and *BoCRTISO1b* (Table 2). The comparison between carotene pathway cDNAs isolated in the Jako and co-workers library [14] showed that only the cDNAs encoding *BoPSY1*, *BoPSY2*, *BoPDS1* and *BoZDS* were in common (Table 2).

In order to elucidate the evolutionary relationship of MEP and carotenoid pathways enzymes from *B. orellana* and other plant species, we carried out a phylogenetic analysis using MEGA6 software. The analysis was based on alignment of concatenated protein sequences from MEP and carotenoid pathways of *B. orellana* and 27 plants species. *B. orellana* was grouped with species from the Malvidae clade and was closely related to cotton and cacao, the two Malvales species available in sequence databases (Fig. 2b).

### Identification of new genes in bixin pathways

To identify and isolate the cDNAs encoding enzymes of the bixin pathway, a TBLASTN search against the achiote transcriptome was performed using the achiote protein sequences previously reported by Bouvier and co-workers (BoLCD, [GenBank: AJ489277]; BoBADH, [GenBank: AJ548846]; BonBMT, [GenBank: AJ548847]) [12]. Surprisingly, these three proteins were not present among those encoded by our assembled transcriptome. The Bouvier BoLCD protein had only one hit with 53 % of identity. BoBADH displayed hits with seven contigs with low identity percentages (49–52 %). When BonBMT was compared, several hits with identity range between 35 and 49 % were found. On the other hand, our previously described CCD1 [13] matched several contigs with high identity (75–98 %). We were also able to identify high quality matches in *B. orellana* for cDNAs encoding carotenoid cleavage dioxygenase 4 (CCD4), aldehyde dehydrogenases (ALDHs) and carboxyl methyltransferases using homologous proteins of *A. thaliana* and *T. cacao*.

#### Carotenoid cleavage dioxygenase proteins in bixin synthesis

The contigs similar to the CCD1 isolated by Rodríguez-Ávila and co-workers [13], allowed the identification of three paralogous copies of the CCD1 gene (*BoCCD1-2*, *BoCCD1-3* and *BoCCD1-4*). A pair-wise comparison between CCD1 protein sequences showed that the BoCCD1 described by Rodríguez-Ávila and co-workers [13] shared 96.9 % identity with BoCCD1-2, 75 % with BoCCD1-3 and 75 % with BoCCD1-4 (Additional file 1: Table S5). Additionally, another CCD1 sequence was identified by PCR when *BoCCD1-2* sequences were amplified and characterized for corroboration. This new cDNAs probably corresponds to an allele of *BoCCD1-2* because it shared 97 % of nucleotide identity. The gene was called *BoCCD1-1.* BoCCD1-1 protein shared 98 % identity with the CCD1 isolated by Rodríguez-Ávila and co-workers [13] and 95 % with BoCCD1-2 (Additional file 1: Table S5). No *BoCCD1* genes were reported by Jako and co-workers (Table 2) [14]. Comparison of CCD4 homologous proteins against those encoded by the assembled achiote transcriptome allowed us to identify five *BoCCD4* genes (*BoCCD4-1*, *BoCCD4-2*, *BoCCD4-3*, *BoCCD4-4*, and *BoCCD4-5*). The pair-wise comparison between these proteins exhibited an identity range between 47 to 67 % (Additional file 1: Table S5). The previous CCD4 isolated by Bouvier and co-workers [12] displayed low identity (30-35 %) in comparison with the proteins coded by our transcriptome (Additional file 1: Table S5). Of the five *BoCCD4* cDNAs characterized in this work, *BoCCD4-2* and *BoCCD4-3* matched EST sequences from Jako and co-workers library (Table 2) [14].

Phylogenetic analysis of BoCCDs proteins yielded two major clades; BoCCD1 and BoCCD4 clustered with the CCD1 and CCD4 families, respectively. BoCCD1-1 and −2 were closely related to the BoCCD1 described by Rodríguez-Ávila and co-workers [13]. BoCCD1-1 and −2 clustered with monocotyledonous CCD1 proteins, albeit with poor bootstrap support. BoCCD1 copy 3 and copy 4 were not closely related to the BoCCD1 protein described by Rodríguez-Ávila and co-workers [13], but grouped together outside the major CCD1 clade (Additional file 2: Figure S1). With regard to the BoCCD4 proteins, BoCCD4-1, −2, −3 and −4 are grouped together (Additional file 2: Figure S1). The small BoCCD4 family clustered in a subclade of CCD4 proteins from woody plants such as *T. cacao*, *Vitis vinifera*, and *Populus trichocarpa*. The incomplete sequence of BoCCD4-5, suggests a more distant relationship to the BoCCD4 small family defined by the previous proteins. BoCCD4-5 is related to the CCD4 from *Ricinus communis*, *P. trichocarpa*, *T. cacao* and *G. raimondii* grouped in the other CCD4 subclade (Additional file 2: Figure S1). The BoLCD sequence described by Bouvier and co-workers [12] was not closely related to BoCCD4 proteins found in this work, but grouped instead in the monocotyledonous CCD4 clade, close to three CCD4 from monocotyledonous *Crocus sativus* (Additional file 2: Figure S1). This latter clade’s strong support (99 % bootstrap value) suggests that their previous attribution to *B. orellana* by Bouvier and co-workers [12] is spurious.

#### Aldehyde dehydrogenase proteins

To identify cDNAs encoding BoALDHs, we performed TBLASTN search using *T. cacao* and *A. thaliana* homologous ALDH proteins from the 13 distinct ALDH families of plants. This approach succeeded in identifying 20 different ALDHs cDNAs. According to the phylogenetic analysis of BoALDH and its homologous proteins, the BoALDHs isolated in this work belong to 10 ALDH families (Table 2 and Additional file 2: Figure S2). Four BoALDH proteins were clustered in the ALDH2 family, five with ALDH3, three with ALDH6 and two with ALDH18. The remaining BoALDH proteins grouped with the ALDH5, ALDH7, ALDH10, ALDH11, ALDH12 and ALDH22 families (Table 2 and Additional file 2: Figure S2). BoBADH described by Bouvier and co-workers [12] was more distant to BoALDHs, and closer to the protein from monocotyledonous *Crocus sativus* in subfamily ALDH2C4 (Additional file 2: Figure S2), another possible spurious instance. BoALDH3H1-1, BoALDH3I and BoALDH7B4 genes yielded BLAST hits with 10, 2 and 1 sequences respectively in the Jako and co-workers library [14] (Table 2).

#### Methyltransferases proteins

In order to identify carboxyl methyltransferase proteins encoded by *B. orellana* transcriptome, we used *T. cacao* and *A. thaliana* homologous proteins belonging to the SABATH methyltranferase family (plant proteins with the ability to methylate carboxyl groups [23]) to perform a TBLASTN search. We found 12 different proteins (Table 2 and Additional file 2: Figure S3). Phylogenetic analysis of SABATH proteins divided them in three major clades called I, II and III (Additional file 2: Figure S3), which, however, differed from a previous phylogenetic classification [23]. BoSABATH1, BoSABATH2 and a small group of four BoSABATH proteins (BoSABATH 3, 4, 5 and 6) were grouped in Clade I. Also, the previously described BonBMT was grouped in this clade, but was not closely related to our BoSABATH protein. Instead, it displayed high similarity to a *C. sativus* carboxyl methyltransferase. This clade’s strong support (96 % bootstrap value) suggests another spurious instance of BonBMT described by Bouvier and co-workers [12] (Additional file 2: Figure S3). BoSABATH2 was the only one grouped in the small clade II, for which most members are jasmonic acid carboxyl methyltransferases. In clade III, BoSABATH10 was grouped in a subclade formed by ten *A. thaliana* SABATH proteins. Additionally, BoSABATH7, 8, 9, 11 and 12 were clustered in clade III and a small BoSABATH group was formed by BoSABATH8, 11 and 12 (Additional file 2: Figure S3). *BoSABATH3* and *BoSABATH4* proteins matched, respectively, 3 and 6 sequences in Jako and co-workers library [14] (Table 2).

### Gene expression of selected carotenoid and bixin pathway key genes

**Fig. 6:**
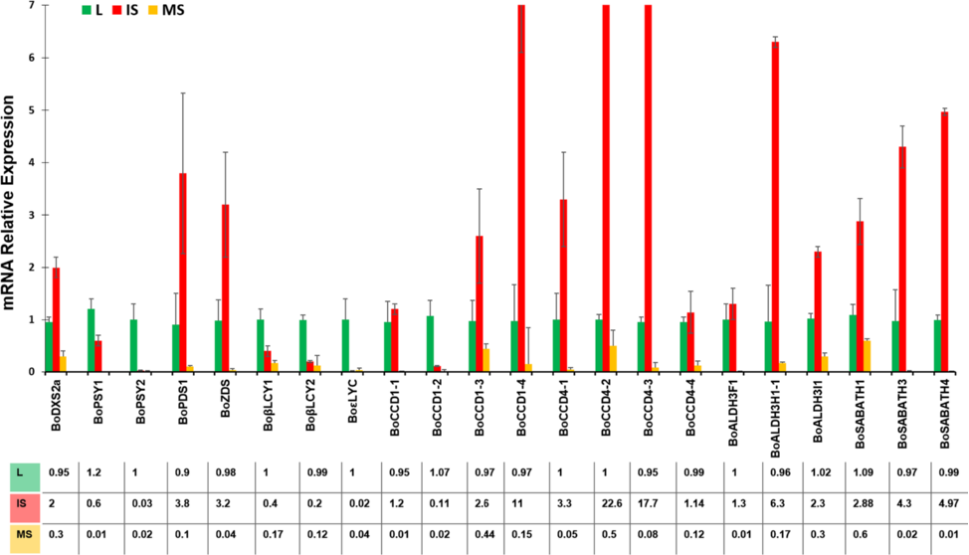
qRT-PCR quantification. Quantitative analysis by qRT-PCR of selected genes encoding enzymes involved in MEP, carotenoid and bixin biosynthesis in leaves (L), immature seeds (IS), and mature seeds (MS) of *Bixa orellana*. The relative mRNA levels were normalized according to a control gene (18S ribosomal) and expressed relative to the corresponding values of leaf (reference sample). Reported values represent means ± SD (standard deviation) of three independent biological replicates

We selected key cDNAs of the carotenoid and bixin biosynthesis pathways for qRT-PCR quantification of their transcript levels in new RNA samples from leaves, immature seeds and mature seeds (Fig. 6). In the MEP pathway, we found that *BoDXS2a* was overexpressed in immature seed in comparison to mature seed and leaf (Fig. 6). In the carotenoid pathway, we select *BoPSY1*, *BoPSY2*, *BoPDS1, BoZDS Boβ-LYC1*, *Boβ-LYC2* and *Boε-LYC* for qRT-PCR quantification. *BoPDS1* and *BoZDS* were up-regulated in immature seed whereas *BoPSY1*, *BoPSY2, Boβ-LYC1*, *Boβ-LYC2* and *Boε-LYC* were expressed preferentially in leaf (Fig. 6). In the bixin pathway, we selected 14 cDNAs, four *BoCCD1* (*BoCCD1*-1 to −4), four *BoCCD4* (*BoCCD4*-1 to −4), three *BoALDH3* (*BoALDH3F1*, *BoALDH3H1* and *BoALDH3I1*) and three *BoSABATH* (*BoSABATH1*, *BoSABATH3* and *BoSABATH4*). *BoCCD1-1*, *BoCCD4-4* and *BoALDH3F1* displayed no changes in transcript levels between leaf and immature seed, whereas the remaining genes showed differential expression levels. Amongst these differential expressed genes, ten were up-regulated in immature seeds and one was up-regulated in leaves (*BoCCD1-2*) (Fig. 6). In all cases the lowest expression levels were displayed in mature seed (Fig. 6). The oligonucleotides sequences used as primers are listed in Additional file 1: Table S6.

## Discussion

Achiote plants are the source of bixin apocarotenoid. Therefore, identification in this species of the genes encoding the putative enzymes of the pathways contributing to bixin synthesis, such as MEP, carotenoid and bixin pathways, is of basic and applied importance. Description of these genes before this study was limited and incomplete [12–15, 24, 25], probably due to coverage limitation of the available EST libraries from immature seeds [14, 25]. A complicating factor is that *B. orellana* is recalcitrant to molecular biology studies, probably because its tissues contain high amounts of secondary metabolites that hinder purification of nucleic acids [16]. With development of high throughput sequencing technology, which are effective with lesser amounts and shorter fragments of RNA, whole transcriptome sequencing became feasible in *B. orellana*. This technology has successfully been applied to identify the MEP and carotenoid pathways genes in *Momordica cochinchinensis* [26], *Citrus sinensis* [27] and *Citrullus lanatus* [28]. Application of this technology to sequencing the first *B. orellana* transcriptome allowed us to elucidate the complete bixin biosynthesis pathway including MEP and carotenoid pathways.

### Transcriptome assembling of *Bixa orellana*

A total of 52,549 contigs were obtained from the transcriptome assembly, which was carried out with the combined use of three assembly programs, Velvet, CLC and CAP3, each providing complementary strengths [19]. A total of 25,555 proteins larger than 100 aa were predicted in the achiote transcriptome, a number similar to that of other sequenced species such as *T. cacao*, *C. papaya*, *C. sinensis*, *C. clementina* and *V. vinifera* [29–32]. BLAST comparison of this transcriptome with the existing *B. orellana* library database [14] and 21 homologous proteins previously isolated [12, 13, 33–37], confirmed that our *B. orellana* assembly is reliable because of high coverage and identity (Additional file 1: Table S2 and Table S7). Moreover, the cDNA sequence covering predicted full length ORFs of carotenoid (*BoPSY1*, *BoPSY2*, *BoPDS1*, *BoZ-ISO*, *BoZDS*, *BoCRTISO1*, *BoCRTISO2* and *BoβLYC*1) and bixin (Five *BoCCD1s* and four *BoCCD4s*) pathways genes obtained through the *in silico* assembly were confirmed by independent cDNA sequencing.

### Evolutionary relationship of *Bixa orellana*

According to the Angiosperm Phylogeny Group (APG) system, *B. orellana* belongs to the Malvales order, Malvidae clade. Malvales include several commercial crops such as kenaf (*Hibiscus cannabinus*), roselle (*Hibiscus sabdariffa*), cacao (*Theobroma cacao*), cotton (species of *Gossypium*) and cola nut (*Cola acuminata*) [1, 2]. Phylogenetic reconstructions based on two sets of *B. orellana* proteins (13 general proteins encoded by single copy genes [21] and additional selected proteins of the carotenoid/MEP pathways) is in agreement with APG classification. As shown in Fig. 2, *B. orellana* is grouped with two members of Malvales available in sequence databases (*T. cacao* and *G. raimondii*). Interestingly, this small group is more closely related to members of the order Malpighiales (*R. communis M. esculenta* and *P.trichocarpa*) than to other orders of Malvidae such as Brassicales or Huertelaes. This discrepancy has been documented, suggesting that the order Malpighiales belongs to the Malvidae rather than Fabidae [38, 39]. The evolutionary relationship of *B. orellana* with Malvales and Malpighiales is also reflected in the comparison of the whole achiote transcriptome against plant protein databases (Fig. 3). As shown in Fig. 3 cacao is most represented among the matches in the Phytozome and Plaza 3.0 comparisons, followed by cotton (*G. raimondii*), cassava (*M. esculenta)*, citrus (*C. sinensis),* poplar (*P. trichocarpa),* papaya (*C. papaya)* and castor bean (*R. communis).* Comparison to RefSeq was biased because most proteins of *G. raimondii*, *M.esculenta* and *C. papaya* were not available there through May, 2014.

### Methylerythritol phosphate (MEP) pathway genes

**Fig. 7:**
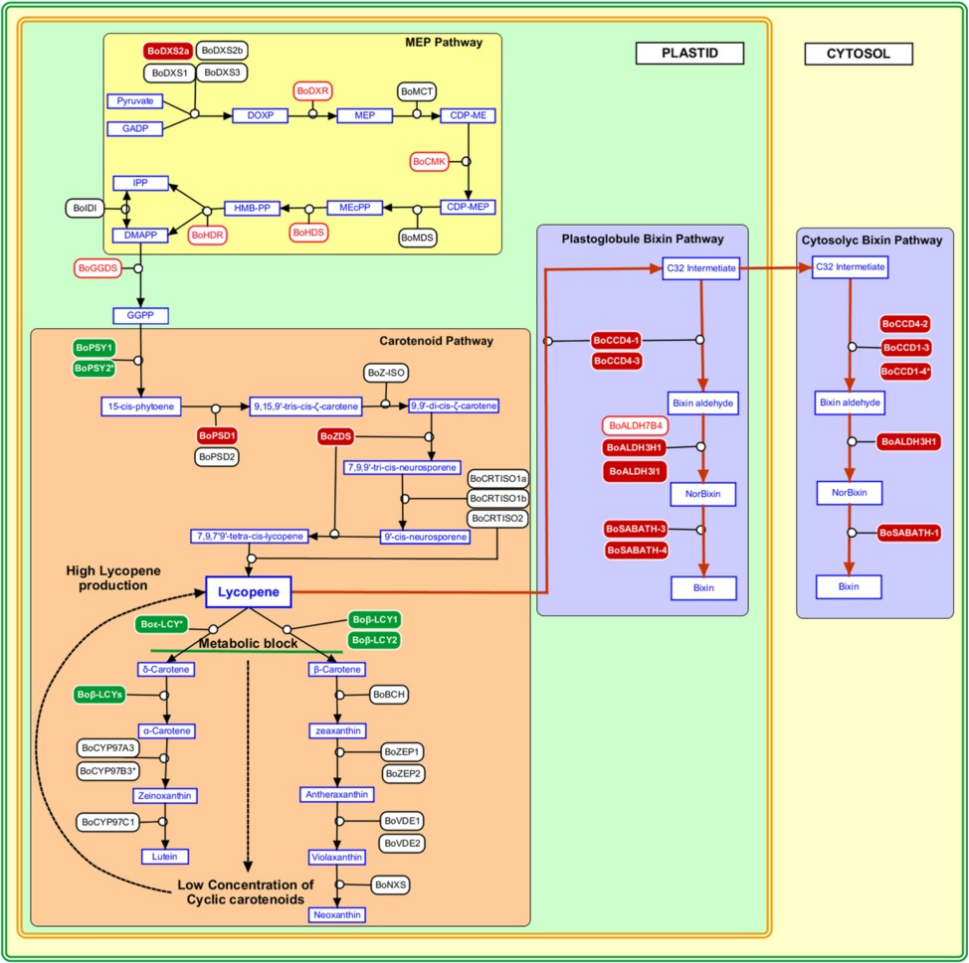
Model of gene regulation in bixin biosynthesis. Genes with qRT-quantification are represented with filled rectangles. Filled red rectangles indicate genes displaying increased expression in immature seed. Filled green rectangles indicate downregulated genes. Red unfilled rectangles indicate genes represented in the Jako’s immature seed library. Asterisks denote partial sequences. The green line indicates blocked downstream process. The green square represents the plastid. The yellow square represents the cytosol. Bright yellow marks the MEP pathway genes. The orange square contains the carotenoid pathway genes and the blue square the bixin pathway. The dashed arrow indicates lycopene feedback regulation. The figure was generated with PathVision 3.1.3 [80]

The MEP pathway is the predominant supplier of carotenoid biosynthesis precursors isopentenyl and dimethylallyl diphosphate (IPP and DMAPP) [40]. In this pathway, pyruvate and glyceraldehyde 3-phosphate are condensed and converted to IPP and DMAPP by seven enzymes (DXS, DXR, MCT, CMK, MDS, HDS and HDR). In this work, we identified the genes encoding these enzymes (Table 2 and Fig. 7). Similar to species with multi-copies of DXS gene [28, 41, 42], achiote also has a small family of four *BoDXS* genes. Phylogenetic analysis of DXS proteins grouped one protein in the DXS type I clade (BoDXS1), two proteins in the DXS type II clade (BoDXS2a and BoDXS2b) and the last (BoDXS3) in the DXS type III clade (Additional file 2: Figure S4). Enzymes from the DXS2 clade, but not DXS1 or DXS3, are involved in carotenoid and apocarotenoid accumulation in non-photosynthetic tissues like seeds [41, 43, 44]. In this work, we found that the *BoDXS2a* gene was overexpressed in immature seeds (Fig. 6), which suggests that BoDXS2a could be involved in the synthesis of seed carotenoids and apocarotenoids. Overexpression in immature seed of *BoDXS2a* (this work)*,* and *BoDXR*, *BoHDS* and *BoHDR* (Table 2) [14], might lead to high concentration of carotenoids and apocarotenoids in immature seed.

### Carotenoid pathway genes of *Bixa orellana*

The carotenoid biosynthetic pathway includes 14 enzymes that convert two GGDP molecules into a variety of carotenoids. Here, we infer from cDNA characterization the existence of 21 genes encoding these enzymes (Table 2 and Fig. 7). With the exception of *BoPSY*, the qRT-PCR quantification profiles suggest enhanced lycopene production in immature seeds, analogous to what was observed during red ripening in tomato fruits. The accumulation of lycopene in tomato is apparently due to downregulation of *β-LYC* and *ε-LYC,* and upregulation of *PSY*, *PDS* and *ZDS* [45–49]*.* Positive feedback regulation may occur during tomato ripening: expression of *PDS* and *ZDS* increases in response to low quantities of end-products of the carotenoid pathway, such as β-carotene, xanthophylls or ABA [49, 50]. A similar scenario could take place in immature seed of *B. orellana*: genes that encode cyclase enzymes were down-regulated in immature seed (Fig. 6), potentially blocking the carotenoid pathway below lycopene and leading to a decrease in cyclic carotenoids concentration. *BoZDS* and *BoPDS1* overexpression in immature seed (Fig. 6) could thus be a response to low concentrations of end-products in the carotenoid pathway (Fig. 7). Consistent with such a block at the immature seed stage, low β-carotene and ABA levels [13] correlated with the presence of *PDS* and absence of lycopene cyclase transcripts (*β-LYC* and *ε-LYC*) in this tissue [15]. If this block is occurring, the lycopene could accumulate in immature seeds increasing the availability of this compound for the bixin pathway. In conclusion, these results are consistent with the hypothesis that lycopene is the main precursor of bixin [12–14].

### Identification of new candidates Bixin biosynthesis pathway genes

Bixin is an orange-red apocarotenoid that accumulates in high quantities in seeds, accounting for 80 % of the total carotenoids. Concentrations of bixin increase continuously during development of immature seeds until they reach maximum size [13]. How is lycopene converted into bixin? The literature indicates the action of three types of enzymes: 1. Carotene cleavage deoxygenase; 2. Aldehyde dehydrogenase; and 3. Methyltransferase. Putative *B. orellana* sequences encoding these enzymes have been described [12]. Surprisingly, we were unable to find transcripts corresponding to the sequences proposed for the above enzymes. Instead, we identified mRNAs encoding different BoCCDs, BoALDH and BoMTs enzymes and believe that these are involved in bixin synthesis. The discrepancy between these and previous findings is explained by the phylogenetic placements of these proteins. The enzymes proposed by Bouvier and co-workers [12] are placed in clades corresponding to monocotyledonous species such as *Crocus sativus*. Furthermore, BoLCD and BonBMT placement in these clades is well supported with bootstrap values of 99 and 96 %, respectively (Additional file 2: Figure S1-S3). It is therefore likely that these cDNAs are not from *Bixa orellana*, but may have been misplaced in the original study. The sequences proposed here for these enzymes, on the other hand, are in the same phylogenetic branch as cotton, cacao and other dicotyledonous plants and were confirmed as Bixa sequences by PCR amplification using independent *Bixa orellana* RNA samples.

#### Carotenoid cleavage dioxygenase candidate proteins in bixin synthesis

The initial step of bixin synthesis is the 5-6/5′-6′ oxidative cleavage of lycopene catalyzed by carotenoid cleavage oxygenase to produce bixin aldehyde [12, 14]. In plants, nine types of carotenoid cleavage dioxygenase have been identified, but only the CCD type 1 and type 4 have been associated with pigment pathways [12, 51–54]. We identified nine putative CCD proteins, four of them CCD type 1 and five type 4 (Table 2 and Additional file 2: Figure S1). As can be seen in Additional file 2: Figure S1, BoCCD1-1 and BoCCD1-2 were closely related to previously isolated CCD1 [13] and they are grouped with monocotyledonous BoCCD1 proteins; this cluster, which was also present in other phylogenetic analysis of CCD family [55], is not well supported with a bootstrap values of 11 in this study and 67 [55], and could be spurious. The gene expression level of previously isolated *BoCCD1,* correlated with bixin accumulation in *B. orellana* [13]. This suggests that *BoCCD1-1* and *BoCCD1-2* could be involved in the cleavage of carotenes to produce seed apocarotenoids, such as ABA and bixin. However, our qRT-PCR analysis indicated that *BoCCD1-1* is equally expressed in leaf and immature seed. *BoCCD1-2* was preferentially expressed in leaf. Unlike these genes, *BoCCD1-3* and *BoCCD1-4*, were overexpressed ~1.5 times and ~10 times in immature seed compared to leaf, respectively (Fig. 6). This suggests that *BoCCD1-3* and *BoCCD1-4* are involved in the cleavage of carotenes in immature seed. CCD1 enzymes have the ability, *in vitro*, to cleave the 5-6/5′-6′ bond in acyclic carotenoids like lycopene (reviewed in [10]). However, experimental subcellular localizations of CCD1 proteins indicated that they are localized in the cytosol without direct access to lycopene [54, 56]. *In silico* prediction of protein properties suggests that BoCCD1-3 is not localized in the chloroplast and presumably does not have direct access to lycopene (Additional file 1: Table S8), therefore it could not be involved in the bixin pathway unless it cleaves lycopene in the cytosol.

CCD4 has the ability to cleave lycopene at the 5, 6/5′,6′ double bond position and the enzymatic activity is specifically associated with plastoglobules within plastids where it has access to its carotenoid substrates [12, 53, 57–59]. We assembled four cDNAs that were each predicted to encode a complete *BoCCD4* ORF (Copy 1–4). The small family formed by these four proteins (Additional file 2: Figure S1) probably originated by duplication, as it appears to be present in other woody plants like *T. cacao* and *P. trichocarpa*. qRT-PCR quantification indicated that *BoCCD4-1*, *BoCCD4-2* and *BoCCD4-3* were upregulated in immature seed, suggesting their involvement in the first step of the bixin pathway (Fig. 6). The cDNAs encoding the BoCCD4-2 and BoCCD4-3 proteins were also represented in the previous immature seed library (Table 2) [14]. According to subcellular localization prediction, BoCCD4-1 and BoCCD4-3 are localized in chloroplasts, whereas BoCCD4-2 is localized in the cytosol (Additional file 1: Table S8). Taken together, this evidence suggests that BoCCD4-1 and BoCCD4-3 cleave lycopene in plastids, where bixin is synthesized. We cannot dismiss the possibility that BoCCD1-3 and BoCCD4-2 could participate in the first step of bixin synthesis. Alternatively, the bixin pathway could be localized both in plastids and in the cytosol. In this case, BoCCD4-1 and BoCCD4-3 could cleave one 5–6 lycopene double bound in plastids followed by export of the resulting C_32_ intermediate to the cytosol. Next, BoCCD1-3 and BoCCD4-2 would cleave the other 5′-6′ double bond to produce bixin aldehyde, and cytosolic BoALDHs and BoSABATH would complete the bixin pathway (Fig. 7). The sequential cleavage, first in plastid and then in cytosol, has been demonstrated in the mycorradicin pathway [60, 61].

#### Aldehyde dehydrogenase candidate proteins in bixin synthesis

The second step in the bixin pathway is the oxidation of aldehyde groups in bixin aldehyde, into carboxylic acids by aldehyde dehydrogenase [12, 14]. Thirteen distinct families of plant aldehyde dehydrogenases enzymes have been identified, although only ten families (ALDH2, 3, 5, 6, 7, 10, 11, 12, 18 and 22) are present in most plant species [62]. Previously identified *B. orellana* ALDHs that could be involved in the bixin pathway include five clusters of ESTs differentially expressed in immature seed [14], and one BoBADH [GenBank: AJ548846] [12], which appears to be a member of the ALDH2 family, specifically type 2C_4._ BoBADH is related to ALDH2C4 of monocotyledonous plants, especially that of *C. sativus* (Additional file 2: Figure S2). Here, we identified 20 *BoALDH*s cDNAs from the ten families constituting the common core group (Table 2 and Additional file 2: Figure S2). A partial *BoALDH2C4* sequence was also identified in the transcriptome. The fact that ALDH2C4 isolated by Bouvier and co-workers [12] is capable of converting aldehyde groups from bixin aldehyde into carboxylic acids and that it is predicted to localize in the chloroplast (Additional file 1: Table S8), suggests that BoALDH2C4 could catalyze the second step of the bixin pathway in plastids. Alternatively, BoALDH2C4 could be acting in the cytosol because in silico prediction and experimental data indicate that orthologous *A. thaliana*, *G. max*, *Z. mays*, *E. parvula* and *E. salsugineum* ALDH2C4 proteins have cytosolic localization [63–66].

Based on subcellular localization prediction, qRT-PCR quantification and presence in the Jako’s library [14], the other three BoALDH (BoALDH3H1-1, 3I1, and 7B4) could also be involved in the bixin pathway. The subcellular localization predicted by Plant-mPLoc and PLpred for BoALDH3H1-1, BoALDH3I1 and BoALDH7B4 indicate that they are localized in chloroplast, where they could have access to bixin aldehyde (Additional file 1: Table S8). Additionally, orthologous proteins predicted to be localized in the chloroplast are found in *A. thaliana*, (ALDH3I1), *Zea mays* (ALDH3H1), *E. parvula* and *E. salsugineum* (ALDH3H1 and ALDH3I1), and *G. max* (ALDH7B4) [64, 65, 67, 68]. *BoALDH3H1-1*, *BoALDH3I1* and *BoALDH7B4* are found in the immature seed Jako’s library [14]. Moreover, our qRT-PCR analyses indicate that *BoALDH3I1* and *BoALDH3H1-1* are also upregulated in immature seed (Fig. 6). The subcellular localization of these three proteins in immature seed and the broad range of substrates catalyzed, suggest that these proteins could catalyze the second step in bixin pathway to produce norBixin in plastid or cytosol. The best candidates for this role, however, are BoALDH3I1 and BoADLH3H1 because these enzymes can act on various substrates in plastids (BoADLH3H1 and BoALDH3I1) or cytosol (BoADLH3H1) (Additional file 1: Table S8) [67]. Moreover, orthologous ALDH3H1 and ALDH3I1 proteins from *Synechocystis* sp. (SynAdh1), *Neurospora crassa* (YLO-1) and *Fusarium fujikuroi* (carD) have the ability to oxidize aldehyde groups from apocarotenoides into carboxylic acids [69–71].

#### Methyltransferases candidate proteins in bixin synthesis

The last step of bixin biosynthesis involves a methyltransferase that methylates a norBixin carboxyl group; members of the SABATH methyltransferase family methylate carboxyl groups [23]. This family also includes enzymes that methylate nitrogen atoms. Previous SABATH methyltransferases identified in *B. orellana* include two clusters of ESTs from the Jako’s library [14], and BonBMT, which methylates the carboxyl groups of norBixin (GenBank: AJ548847) [12]. Here, we identified 12 SABATH methyltransferases. None of them is closely related to BonBMT (Additional file 2: Figure S3), which is grouped with the *C. sativus* methyltransferase. BoSAMTH1, 3, 4, 5 and 6 are placed in the same clade, raising the possibility that these proteins share the function of methylating norBixin. In this group of proteins, BoSABATH1 could be involved in bixin synthesis because qRT-PCR indicated that it is overexpressed in immature seed (Fig. 6). Probably, BoSABATH1 methylates norBixin in the cytosol because it is not predicted to have a plastidial localization (Additional file 1: Table S8). qRT-PCR analysis of *BoSABATH3* and *BoSABATH4* transcripts shows that they are upregulated in immature seed (Fig. 6), thus suggesting that these proteins could be involved in bixin biosynthesis; furthermore, these proteins are represented in the Jako’s immature seed library [14]. Subcellular localization prediction indicates that BoSABATH3 and BoSABATH4 are plastidial proteins with direct access to norBixin in chloroplast or chromoplast. Additionally, we identified 26 methyltransferases involved in secondary metabolism (data not shown), but these were not taken into consideration as candidates for norBixin methylation because most methylate oxygen atoms in benzenic rings.

### Bixin biosynthesis model

Bixin production involves the coordinate expression of the MEP, carotenoid and bixin pathways genes in immature seed. Figure 7 illustrates three molecular steps necessary to synthetized bixin: 1. *BoDXS2a* and others MEP genes involved in generation of carotenoids precursor such as *BoDXR* and *BoHDR* are induced to produce carotenoids in non photosynthetic tissue. 2. Lycopene cyclase genes (*Boβ-LYC1*, *Boβ-LYC1* and *Boε-LYC*) are turned off, thus blocking metabolic flow toward cyclic carotenoids downstream of lycopene. The low concentrations of β-carotene and xanthophyll, induce the expression of *BoPDS1* and *BoZDS* and promote lycopene production in plastoglobules of immature seed cells. In this scenario, also PSY should be upregulated, as suggested by its representation in the Jako’s library [14]. Surprisingly, the two genes found in this transcriptome were downregulated in our dataset. 3. The *BoCCDs* (*BoCCD1-3, BoCCD1-4, BoCCD4-1*, *BoCCD4-2* and *BoCCD4-3*), *BoALDH3* (*BoALDH3H1* and *BoALDH3I1*) and *BoSABATH* (*BoSABATH1*, *BoSABATH3* and *BoSABATH4*) genes are then turned on leading to lycopene conversion to bixin in plastoglobules or cytosol (Fig. 7).

## Conclusion

Deep sequencing of the *Bixa orellana* transcriptome enabled the the isolation and characterization of the complete MEP and carotenoid pathway genes. Our inability to find in this transcriptome cDNAs previously identified by Bouvier and co-workers [12], lead us to propose new and alternative enzymes, whose identification was based on the upregulation of the corresponding genes. These findings will help elucidate the regulatory mechanisms controlling the production and accumulation of carotenoid and bixin in *B. orellana.* For this, characterization of the enzymatic activities proposed here will be necessary. Finally, this information will help identify the candidate genes and mechanisms for variation of apocarotenoids accumulation in achiote varieties, thus facilitating the genetic improvement of achiote for high bixin content.

## Methods

### Plant material and total RNA isolation

Samples of young leaves, immature and mature seeds were harvested from *B. orellana* plants cultivated at a commercial plantation in Chicxulub, Yucatán, Mexico. All tissues were obtained from a *B. orellana* accession “Peruana Roja”, a variety with pink flowers and high pigment contents characterized by Rivera-Madrid and co-workers [72] (Fig. 1). The fresh tissues were immediately frozen in liquid nitrogen and stored at −80 °C until analysis. Total RNA was isolated from leaves, immature and mature seeds from *B. orellana*, accession PR, according to the protocol of Rodríguez-Ávila and co-workers [16].

### Illumina sequencing and *de novo* assembly

Total RNA from the different tissue was used for the construction of indexed mRNA libraries using KAPA Stranded mRNA-Seq Kit Illumina platform (KAPA Biosystems: KR0960). Libraries were paired end sequenced with 150 cycles in two lanes of the Illumina HiSeq 2500 platform (~300 million reads total) using two insert sizes: 250 bp for read overlap, and 450 bp for paired reads. The long reads are necessary for the assembly of homologous sequences. Reads were then demultiplexed and preprocessed for quality using scripts developed by the Comai laboratory and available online (http://comailab.genomecenter.ucdavis.edu/index.php/Barcoded_data_preparation_tools). Reads were trimmed for quality when the average Phred sequence quality over a 5 bp window dropped below 20, trimmed for adapter sequence contamination, and discarded if the final length was shorter than 35 b. For the assembly process reads were processed through the Velvet assembler [17], using kmer sizes ranging from 21 to 53 and a range of expected coverages. The same read set was then also put through CLC Genomics Workbench *de novo* assembler (http://www.clcbio.com). The Velvet assemblies had duplicates removed and then was combined with the CLC contig set. This combined contig set was reduced to contigs in the size range of 300 bp – 10 kbp, and was then put into CAP3 [18] to create transcript contigs. Assemblathon2 Perl script [73] were used to compute assembly statistics. As demonstrated by Ashrafi [19] Velvet and CLC assembly algorithms were found to have complementary qualities for the initial assembly. CAP3 was used as a superassembler to extend Velvet and CLC contigs.

### Blast search in public databases

A local BLAST analysis was performed to compare the achiote transcriptome (52,549 contigs) with three protein databases, NCBI Plant Protein Reference sequence (RefSeq) update in May, 2014, Phytozome v10.0.2 and PLAZA 3.0. The BLASTX algorithm included in bioinformatics package BLAST+ v2.2.29 [74] was used with an e-value cutoff of 1e-6. In order to compare the transcriptome against a previous *B. orellana* EST library [14], a bidirectional BLASTN analysis with e-value cutoff of 1e-100 was performed. The Jako and co-workers EST library is available in NCBI [GenBank: LIBEST_025681 BIXA] [14].

### Functional annotation

For functional annotation, 52,549 contigs were searched against RefSeq using BLASTX algorithm included in bioinformatics package BLAST+ v2.2.29. The e-value cutoff of 1e-6 was used for the search and 50 alignments were kept. Gene Ontology terms (GO) from GO database (06/may/14) were extracted from BLASTX results using the BLAST2GO program [75]. To get the functional pathway annotation from KEGG pathways in the curated KEGG GENES database, the KAAS tool (KEGG Automatic Annotation Server) was implemented [76].

### Identification of MEP, carotenoid and bixin pathways genes from *B. orellana* transcriptome

Local TBLASTN with e-value cut off of 1e-6 was performed to search the MEP, carotenoid and bixin pathways genes. Homologous protein from *Arabidopsis thaliana*, *Theobroma cacao* and *Gossypium raimondii* were used to make the search against *B. orellana* transcriptome database. If the resultant contigs did not have the complete open reading frame (ORF), then contigs with partial ORFs were isolated and assembled with Lasergen SeqMan software (DNASTAR Inc., Madison, WI, USA).

### Phylogenetic analysis

Phylogenetic reconstruction from proteins codified by a set of 13 single copy genes identified by Duarte and co-workers [21] was based on alignment of concatenated protein sequences from 28 plant species and one moss species. Phylogenetic tree was inferred by the maximum-likelihood method based on Le_Gascuel_2008 (LG) substitution model [77] and Gamma distributed (G). Phylogenetic analysis from MEP/carotenoid enzymes pathways was based on alignment of concatenated sequences from 29 plant species and one moss species. Phylogenetic tree was inferred by maximum-likelihood method based on Jones-Taylor-Thornton (JTT) substitution model [78] and Gamma distributed with Invariant sites (G + I). In both cases the analysis were carried out using algorithms included in MEGA6 [79] and the substitution models were predicted by the Best-Fit substitution model (ML) function included in MEGA6. Phylogeny tests were conducted by the bootstrap method (1000 replicates). All positions containing gaps and missing data were eliminated. The alignments of concatenated sequences were performed with the ClustalW algorithm with default parameters on MEGA6. Phylogenetic trees were rooted with *Chlamydomonas reinhardtii*, a single-cell green alga. Proteins sequences and plant species used are listed in Additional file 1: Table S9.

### Gene expression

The cDNA was synthesized using the SuperScript III First-Strand Synthesis System for the RT-PCR kit (Invitrogen, San Diego, CA) according to the manufacturer’s instructions. After reverse transcription, the cDNAs were amplified by qPCR with 40 cycles and with specific primers (Additional file 1: Table S6). A parallel reaction with 40 cycles and specific primers for the *18S rRNA* gene (5′-CGGCTACCACATCCAAGGAA-3′ and 5′-GCTGGAATTACCGCGGCT-3′, AF206868) was run as an expression control for each PCR reaction. Three replicates of each PCR reaction were carried out to confirm the results. Gene expression relative to the *18S rRNA* gene was assessed using the StepOne Real-Time PCR System (Applied Biosystems catalog number 4376374).

### Availability of supporting data

Supporting data are available in NCBI database.

The *Bixa orellana* transcriptome has been deposited at Transcriptome Shotgun Assembly project at DDBJ/EMBL/GenBank under the accession GDKG00000000. The version described in this paper is the first version, GDKG01000000.

BioProject: PRJNA290519 (http://www.ncbi.nlm.nih.gov/bioproject/290519)

BioSample: SAMN03892718 (http://www.ncbi.nlm.nih.gov/biosample/?term=SAMN03892718)

Sequence Read Archive (SRA): SRR2131178 (http://trace.ncbi.nlm.nih.gov/Traces/sra/sra.cgi?run=SRR2131178)

## Additional files

Additional file 1: Table S1. BLASTX comparison between the *B. orellana* transcriptome against three databases. **Table S2.** BLASTN comparison between the *B. orellana* transcriptome and the previous EST library created by Jako and co-workers [GenBank: LIBEST_025681 BIXA]. **Table S3.** Gene Ontology (GO) annotation. **Table S4.** Kyoto Encyclopedia of Genes and Genomes (KEGG) annotation. **Table S5.** Pairwise comparison between amino acid sequences of carotenoid cleavage dioxygenase proteins. **Table S6.** RT-qPCR primers. **Table S7.** BLASTx comparison between the *B. orellana* transcriptome and previously identified *B. orellana* proteins .**Table S8.** Subcellular localization predictions for the BoCCD, BoALDH and BoSABATH proteins. **Table S9.** Accession number of proteins used in Fig. 1. (ZIP 15364 kb) Additional file 2: Figure S1. Evolutionary relationship of CCDs proteins. **Figure S2.** Evolutionary relationship of ALDH proteins. **Figure S3.** Evolutionary relationship of SABATH methyltransferases proteins. **Figure S4.** Evolutionary relationship of DXS proteins. (ZIP 410 kb)

## Abbreviations

ABA: Abscisic acid
IPP: Isopentenyl diphosphate
MEP: Methylerythritol phosphate
GGDP: Geranylgeranyl diphosphate
PSY: Phytoene synthase
PDS: Phytoene desaturase
ZDS: Zeta-carotene desaturase
CRTISO: Carotene cis-trans isomerase
Z-ISO: ζ-carotene isomerase
ε-LYC: Epsilon-cyclase
β-LYC: Beta-cyclase
CCD: Carotene cleavage dioxygenase
BoLCD: Lycopene cleavage dioxygenase
BoBALDH: Bixin aldehyde dehydrogenase
BonBMT: Norbixin methyltransferase
DXS: 1-Deoxy-D-xylulose-5-phosphate synthase
DXR: 1-Deoxy-D-xylulose-5-phosphate reductoisomerase
MCT: 2-C-Methyl-D-erythritol 4-phosphate cytidyltransferase
HDS: 4-Hydroxy-3-methylbut-2-en-1-yl diphosphate synthase
GGDS: Geranylgeranyl diphosphate synthase
KEGG: Kyoto encyclopedia of genes and genomes
βCH: β-carotene hydroxylase
CYP97A3: Cytochrome P450-type monooxygenase 97A
CYP97C1: Cytochrome P450-type monooxygenase 97C1
ZEP: Zeaxanthin epoxidase
VDE: Violaxanthin de-epoxidase
IDI: Isopentenyl diphosphate isomerase
CMK: 4-Diphosphocytidyl-2-C-methyl-D-erythritol kinase
MDS: 2-C-Methyl-D-erythritol 2,4-cyclodiphosphate synthase
HDR: 4-Hydroxy-3-methylbut-2-enyl diphosphate reductase
RefSeq: NCBI plant protein reference sequence
GO: Gene ontology terms
