# Supplementary material for: De novo transcriptome sequencing in Bixa orellana to identify genes involved in methylerythritol phosphate, carotenoid and bixin biosynthesis
Source: BMC Genomics. 2015 Oct 28;16:877. doi: 10.1186/s12864-015-2065-4 (PMC4625570; doi:10.1186/s12864-015-2065-4)
Supplement: Additional file 1: Table S1. — BLASTX comparison between the B. orellana transcriptome against three databases. Table S2. BLASTN comparison between the B. orellana transcriptome and the previous EST library created by Jako and co-workers [GenBank: LIBEST_025681 BIXA]. Table S3. Gene Ontology (GO) annotation. Table S4. Kyoto Encyclopedia of Genes and Genomes (KEGG) annotation. Table S5. Pairwise comparison between amino acid sequences of carotenoid cleavage dioxygenase proteins. Table S6. RT-qPCR primers. Table S7. BLASTx comparison between the B. orellana transcriptome and previously identified B. orellana proteins .Table S8. Subcellular localization predictions for the BoCCD, BoALDH and BoSABATH proteins. Table S9. Accession number of proteins used in Fig. 1. (ZIP 15364 kb) [file 12864_2015_2065_MOESM1_ESM.zip › Additional file 1_Table S6.docx]

| Table S6. RT-qPCR primers | |
| --- | --- |
| F-DXS2a | CTGCACCATGTCTTCAGCAC |
| R-DXS2a | CTACTGCCATGCCAAGACCA |
| F-PSY1 | GAACTGTGGGGCTAATGAGTGT |
| R-PSY1 | ACTCTTCCTCTCCTAGCATCTTCG |
| F-PSY2 | GTGTAGGAGGACTGATGAGCTTGT |
| R-PSY2 | GCTGAATGTCCACAGGGAAT |
| F-PDS1 | TTGGACTACTGCCAGCAATG |
| R-PDS1 | AAACCGGTTCAAAGCAATCA |
| F-ZDS | AGATCTGGCTCTCACTTCTCC |
| R-ZDS | CTTCTAAGCCTTGAGATGATGG |
| F-βLYC1 | ATTGTCCAGTGCCTTGGTTC |
| R-βLYC1 | CCATGCCAATATCGAGGTTC |
| F-βLYC2 | TTCAAGGCCAGCTTGATCGT |
| R-βLYC2 | GTGTTTGGGTTCGACGAGGA |
| F-εLYC | TTGGAAGTCTTGGAGAAGGAC |
| R-εLYC | ACTTATCGGCTAATCTGGACTG |
| F-CCD1-1 | CTGGCACTTAACGAGGGT |
| R-CCD1-1 | CAACCTTAGGATGAGCAGTG |
| F-CCD1-2 | CTGGCACTTCAAGAGGCA |
| R-CCD1-2 | CAACCTTAGGATGAGCAGTG |
| F-CCD1-3 | TCCCAACCCAAAGTTTCAC |
| R-CCD1-3 | TCCTATGCTTACCATGAGTGG |
| F-CCD1-4 | TGCCAATATGGACGAGTCC |
| R-CCD1-4 | TAGCCATCATCCTCCTCCA |
| F-CCD4-1 | AGCTTCCACCGTCTCTCCA |
| R-CCD4-1 | AATGATCGCAGCTCCTCTGC |
| F-CCD4-2 | GATTCCCACCTCTCTGGA |
| R-CCD4-2 | AACATATTGGGCATGCGA |
| F-CCD4-3 | ATGAGGACACCAAGGACG |
| R-CCD4-3 | CTAGCATCATTTTGGCAACG |
| F-CCD4-4 | TACTGCCAAGATGATCTGG |
| R-CCD4-4 | GCATTGAGGACATGTAATGG |
| F-ALDH3F1 | ATGGAAGAAACCTTGGCAG |
| R-ALDH3F1 | TCAGTTGGGTGCTTTCCT |
| F-ALDH3H1 | TGGTAGATACTTTCAGAGAGG |
| R-ALDH3H1 | GTTGGAGCAATCTTCAGC |
| F-ALDH3I1 | CATTCCACTTCAGACGTC |
| R-ALDH3I1 | TTCTTGCATGATCTGTGC |
| F-SABATH1 | ACGGAGGAAGCTATCACCA |
| R-SABATH1 | GACGTGATATTCAGGGGATC |
| F-SABATH3 | TTATGGCCGTCTCTGAGGT |
| R-SABATH3 | ACCTGGCACTCCTGATACG |
| F-SABATH4 | TCCCGTCTACAAATGGCA |
| R-SABATH4 | AATTTCGGAGATGACCAGG |
