# Supplementary material for: De novo transcriptome sequencing in Bixa orellana to identify genes involved in methylerythritol phosphate, carotenoid and bixin biosynthesis
Source: BMC Genomics. 2015 Oct 28;16:877. doi: 10.1186/s12864-015-2065-4 (PMC4625570; doi:10.1186/s12864-015-2065-4)
Supplement: Additional file 1: Table S1. — BLASTX comparison between the B. orellana transcriptome against three databases. Table S2. BLASTN comparison between the B. orellana transcriptome and the previous EST library created by Jako and co-workers [GenBank: LIBEST_025681 BIXA]. Table S3. Gene Ontology (GO) annotation. Table S4. Kyoto Encyclopedia of Genes and Genomes (KEGG) annotation. Table S5. Pairwise comparison between amino acid sequences of carotenoid cleavage dioxygenase proteins. Table S6. RT-qPCR primers. Table S7. BLASTx comparison between the B. orellana transcriptome and previously identified B. orellana proteins .Table S8. Subcellular localization predictions for the BoCCD, BoALDH and BoSABATH proteins. Table S9. Accession number of proteins used in Fig. 1. (ZIP 15364 kb) [file 12864_2015_2065_MOESM1_ESM.zip › Additional file 1_Table S8.docx]

| **Table S8.** Subcellular localization prediction of BoCCD, BoALDH and BoSABATH proteins | | | | |  |
| --- | --- | --- | --- | --- | --- |
| **Protein** | **PlantmPloc** | **PLpred** | **TargetP** | **Experimentally Confirmed** | |
| **Carotenoid Cleavage Dioxygenase** | | | | | |
| BoCCD1-1 | Cyt | OP | - | *A. thaliana* CCD1-Cytosol [1].  *C. sativus* CCD2-Cytosol [2] | |
| BoCCD1-2 | Cyt | Chl | - |  |  |
| BoCCD1-3 | Cyt | OP | - |  |  |
| BoCCD1-4 | NA | NA | NA |  |  |
| BoCCD4-1 | Cyt | Chl | - | *A. thaliana* CCD4-Chloroplast [3]  *C. sativus* CCD4-Plastoglobule [4] | |
| BoCCD4-2 | Cyt | NP | - |  |  |
| BoCCD4-3 | Cyt | Chl | Chl |  |  |
| BoCCD4-4 | Chl/Cyt | Chl | SP |  |  |
| BoCCD4-5 | NA | NA | NA |  |  |
| BoLCD.Bouvier | Cyt | Chl | - | Not information | |
| BoLCD.Unpublished | Cyt | OP | - | Not information | |
| BoCCD1.Rodriguez | Cyt | OP | - | Not information | |
| **Aldehyde dehydrogenase** | | | | | |
| BoALDH2B4 | Mit | Eti | Mit | *A. thaliana* ALDH2B4-Mitochondria [5] | |
| BoALDH2C4 | NA | NA | NA | *A. thaliana* ALDH2C4-Cytosol [6] | |
| BoALDH2B7-1 | Mit | Chl | Mit | *A. thaliana* ALDH2B7-Mitochondria (Q56YU0) | |
| BoALDH2B7-2 | Mit | Eti | Mit |  |  |
| BoALDH3F1 | Chl | Chl | - | *A.thaliana* ALDH3F1-Cytosol [7] | |
| BoALDH3F2 | Chl | NP | - |  | |
| BoALDH3H1-1 | Chl | Chl | Chl | *A.thaliana* ALDH3H1-Cytosol [7] | |
| BoALDH3H1-2 | Chl | NP | - |  |  |
| BoALDH3I1 | Chl | Chl | - | *A.thaliana* ALDH3I1-Chloroplast [7] | |
| BoALDH5F1 | Mit | Chl | Mit | *A.thaliana* ALDH5F1-Mitochondria [8] | |
| BoALDH6B2-1 | Mit | OP | Mit | *A. thaliana* ALDH6B2-Mitochondria (Q0WM29) | |
| BoALDH6B2-2 | Mit | OP | - |  |  |
| BoALDH6B2-3 | Mit | Chl | - |  |  |
| BoALDH7B4 | Chl | Chl | - | *A. thaliana* ALDH7B4-Cytosol [7] | |
| BoALDH10A8 | Chl/Mit/Per | Eti | - | *A. thaliana* ALDH10A8-Leucoplast [7] | |
| BoALDH11A3 | Cyt | OP | - | *A.thaliana* ALDH11A3-Cytosol (Q1WIQ6) | |
| BoALDH12A1 | Mit | Chl | - | *A. thaliana* ALDH12A3-Mitochondria [9] | |
| BoALDH18B1-1 | Cyt | NP | - |  | |
| BoALDH18B1-2 | Cyt | NP |  |  |  |
| BoALDH22A1 | Chl | OP | - | *A. thaliana* ALDH22A1-Cytosol [7] | |
| BoBADH-Bouvier | Chl | Chl | - | Not information | |
| **SABATH Methyltransferase** | | | | | |
| BoSABATH1 | Cyt/Nuc | NP | - | *A. majus* S-adenosyl-l-methionine:benzoic acid carboxyl methyltransferase-Cytosol [10] | |
| BoSABATH2 | Cyt/Nuc | Chl | - |  |  |
| BoSABATH3 | Cyt/Nuc | Chl | Chl |  |  |
| BoSABATH4 | Cyt/Nuc | Chl | - |  |  |
| BoSABATH5 | Cyt/Nuc | NP | Chl |  |  |
| BoSABATH6 | Cyt/Nuc | Chl | - |  |  |
| BoSABATH7 | Cyt/Nuc | Chl | - |  |  |
| BoSABATH8 | Cyt/Nuc | Chl | - |  |  |
| BoSABATH9 | Cyt | Chl | - |  |  |
| BoSABATH10 | NA | NA | NA |  |  |
| BoSABATH11 | Cyt/Nuc | Chl | - |  |  |
| BoSABATH12 | Cyt/Nuc | Chl | - |  |  |

Cyt: Cytosol; Nuc: Nucleus; Chl: Chloroplast; Mit: Mitochondria; OP: other plastid; NP: Not plastid; Eti: Etioplast; NA: not applied, partial sequence.

1. Auldridge ME, Block A, Vogel JT, Dabney-Smith C, Mila I, Bouzayen M, Magallanes-Lundback M, DellaPenna D, McCarty DR, Klee HJ: **Characterization of three members of the Arabidopsis carotenoid cleavage dioxygenase family demonstrates the divergent roles of this multifunctional enzyme family.** *Plant J* 2006, **45**:982–93.

2. Frusciante S, Diretto G, Bruno M, Ferrante P, Pietrella M, Prado-Cabrero A, Rubio-Moraga A, Beyer P, Gomez-Gomez L, Al-Babili S, Giuliano G: **Novel carotenoid cleavage dioxygenase catalyzes the first dedicated step in saffron crocin biosynthesis.** *Proc Natl Acad Sci U S A* 2014, **111**:12246–51.

3. Ytterberg AJ, Peltier J, Wijk KJ Van: **Protein Profiling of Plastoglobules in Chloroplasts and Chromoplasts . A Surprising Site for Differential Accumulation of Metabolic Enzymes 1 [ W ]**. *Plant Physiol* 2006, **140**(March):984–997.

4. Rubio A, Rambla JL, Santaella M, Gómez MD, Orzaez D, Granell A, Gómez-Gómez L: **Cytosolic and plastoglobule-targeted carotenoid dioxygenases from Crocus sativus are both involved in beta-ionone release.** *J Biol Chem* 2008, **283**:24816–25.

5. Wei Y, Lin M, Oliver DJ, Schnable PS: **The roles of aldehyde dehydrogenases (ALDHs) in the PDH bypass of Arabidopsis.** *BMC Biochem* 2009, **10**:7.

6. Nair RB, Bastress KL, Ruegger MO, Denault JW, Chapple C: **The Arabidopsis thaliana REDUCED EPIDERMAL FLUORESCENCE1 gene encodes an aldehyde dehydrogenase involved in ferulic acid and sinapic acid biosynthesis.** *Plant Cell* 2004, **16**:544–54.

7. Stiti N, Missihoun TD, Kotchoni SO, Kirch H-H, Bartels D: **Aldehyde Dehydrogenases in Arabidopsis thaliana: Biochemical Requirements, Metabolic Pathways, and Functional Analysis.** *Front Plant Sci* 2011, **2**(October):Article 65: 1–11.

8. Bouché N, Fait A, Bouchez D, Møller SG, Fromm H: **Mitochondrial succinic-semialdehyde dehydrogenase of the gamma-aminobutyrate shunt is required to restrict levels of reactive oxygen intermediates in plants.** *Proc Natl Acad Sci U S A* 2003, **100**:6843–6848.

9. Deuschle K, Funck D, Hellmann H, Da K, Binder S, Frommer WB: **A nuclear gene encoding mitochondrial D 1 -pyrroline-5- carboxylate dehydrogenase and its potential role in protection from proline toxicity**. *Plant J* 2001, **27**:345–355.

10. Kolosova N, Sherman D, Karlson D, Dudareva N: **Cellular and Subcellular Localization of S -Adenosyl- L -Methionine : Benzoic Acid Carboxyl Methyltransferase , the Enzyme Responsible for Biosynthesis of the Volatile Ester Methylbenzoate in Snapdragon Flowers 1**. *Plant Physiol* 2001, **126**(July):956–964.
