# Supplementary material for: De novo transcriptome sequencing in Bixa orellana to identify genes involved in methylerythritol phosphate, carotenoid and bixin biosynthesis
Source: BMC Genomics. 2015 Oct 28;16:877. doi: 10.1186/s12864-015-2065-4 (PMC4625570; doi:10.1186/s12864-015-2065-4)
Supplement: Additional file 2: Figure S1. — Evolutionary relationship of CCDs proteins. Figure S2. Evolutionary relationship of ALDH proteins. Figure S3. Evolutionary relationship of SABATH methyltransferases proteins. Figure S4. Evolutionary relationship of DXS proteins. (ZIP 410 kb) [file 12864_2015_2065_MOESM2_ESM.zip › Additional file 2_Figure S4.pptx]

## Slide 1
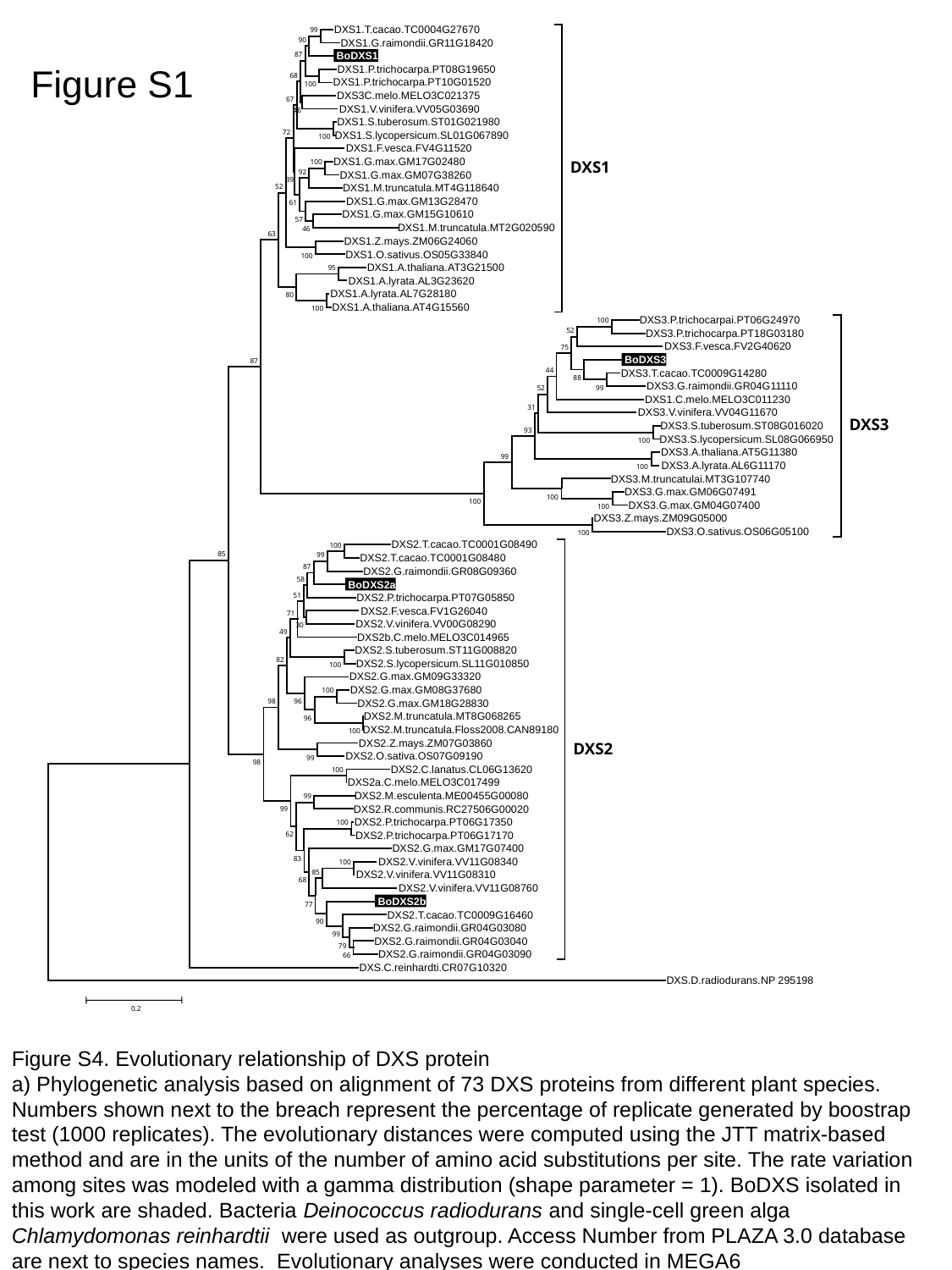

DXS1.T.cacao.TC0004G27670
99
90
 DXS1.G.raimondii.GR11G18420
 BoDXS1
87
 DXS1.P.trichocarpa.PT08G19650
68
 DXS1.P.trichocarpa.PT10G01520
100
 DXS3C.melo.MELO3C021375
67
 DXS1.V.vinifera.VV05G03690
46
 DXS1.S.tuberosum.ST01G021980
72
 DXS1.S.lycopersicum.SL01G067890
100
 DXS1.F.vesca.FV4G11520
 DXS1.G.max.GM17G02480
DXS1
100
92
 DXS1.G.max.GM07G38260
39
 DXS1.M.truncatula.MT4G118640
52
 DXS1.G.max.GM13G28470
61
 DXS1.G.max.GM15G10610
57
 DXS1.M.truncatula.MT2G020590
46
63
 DXS1.Z.mays.ZM06G24060
 DXS1.O.sativus.OS05G33840
100
 DXS1.A.thaliana.AT3G21500
95
 DXS1.A.lyrata.AL3G23620
 DXS1.A.lyrata.AL7G28180
80
 DXS1.A.thaliana.AT4G15560
100
 DXS3.P.trichocarpai.PT06G24970
100
52
 DXS3.P.trichocarpa.PT18G03180
 DXS3.F.vesca.FV2G40620
75
 BoDXS3
87
44
 DXS3.T.cacao.TC0009G14280
88
 DXS3.G.raimondii.GR04G11110
99
52
 DXS1.C.melo.MELO3C011230
31
 DXS3.V.vinifera.VV04G11670
DXS3
 DXS3.S.tuberosum.ST08G016020
93
 DXS3.S.lycopersicum.SL08G066950
100
 DXS3.A.thaliana.AT5G11380
99
 DXS3.A.lyrata.AL6G11170
100
 DXS3.M.truncatulai.MT3G107740
 DXS3.G.max.GM06G07491
100
100
 DXS3.G.max.GM04G07400
100
 DXS3.Z.mays.ZM09G05000
 DXS3.O.sativus.OS06G05100
100
 DXS2.T.cacao.TC0001G08490
100
85
99
 DXS2.T.cacao.TC0001G08480
87
 DXS2.G.raimondii.GR08G09360
58
 BoDXS2a
51
 DXS2.P.trichocarpa.PT07G05850
 DXS2.F.vesca.FV1G26040
71
 DXS2.V.vinifera.VV00G08290
30
49
 DXS2b.C.melo.MELO3C014965
 DXS2.S.tuberosum.ST11G008820
82
 DXS2.S.lycopersicum.SL11G010850
100
 DXS2.G.max.GM09G33320
 DXS2.G.max.GM08G37680
100
 DXS2.G.max.GM18G28830
98
96
 DXS2.M.truncatula.MT8G068265
96
 DXS2.M.truncatula.Floss2008.CAN89180
100
 DXS2.Z.mays.ZM07G03860
DXS2
 DXS2.O.sativa.OS07G09190
99
98
 DXS2.C.lanatus.CL06G13620
100
 DXS2a.C.melo.MELO3C017499
 DXS2.M.esculenta.ME00455G00080
99
 DXS2.R.communis.RC27506G00020
99
 DXS2.P.trichocarpa.PT06G17350
100
 DXS2.P.trichocarpa.PT06G17170
62
 DXS2.G.max.GM17G07400
83
 DXS2.V.vinifera.VV11G08340
100
85
 DXS2.V.vinifera.VV11G08310
68
 DXS2.V.vinifera.VV11G08760
 BoDXS2b
77
 DXS2.T.cacao.TC0009G16460
90
 DXS2.G.raimondii.GR04G03080
99
 DXS2.G.raimondii.GR04G03040
79
 DXS2.G.raimondii.GR04G03090
66
 DXS.C.reinhardti.CR07G10320
 DXS.D.radiodurans.NP 295198
0.2
Figure S1
Figure S4. Evolutionary relationship of DXS protein
a) Phylogenetic analysis based on alignment of 73 DXS proteins from different plant species. Numbers shown next to the breach represent the percentage of replicate generated by boostrap test (1000 replicates). The evolutionary distances were computed using the JTT matrix-based method and are in the units of the number of amino acid substitutions per site. The rate variation among sites was modeled with a gamma distribution (shape parameter = 1). BoDXS isolated in this work are shaded. Bacteria Deinococcus radiodurans and single-cell green alga Chlamydomonas reinhardtii were used as outgroup. Access Number from PLAZA 3.0 database are next to species names.  Evolutionary analyses were conducted in MEGA6
