# Supplementary material for: De novo transcriptome sequencing in Bixa orellana to identify genes involved in methylerythritol phosphate, carotenoid and bixin biosynthesis
Source: BMC Genomics. 2015 Oct 28;16:877. doi: 10.1186/s12864-015-2065-4 (PMC4625570; doi:10.1186/s12864-015-2065-4)
Supplement: Additional file 2: Figure S1. — Evolutionary relationship of CCDs proteins. Figure S2. Evolutionary relationship of ALDH proteins. Figure S3. Evolutionary relationship of SABATH methyltransferases proteins. Figure S4. Evolutionary relationship of DXS proteins. (ZIP 410 kb) [file 12864_2015_2065_MOESM2_ESM.zip › Additional file 2_Figure S1.pptx]

## Slide 1
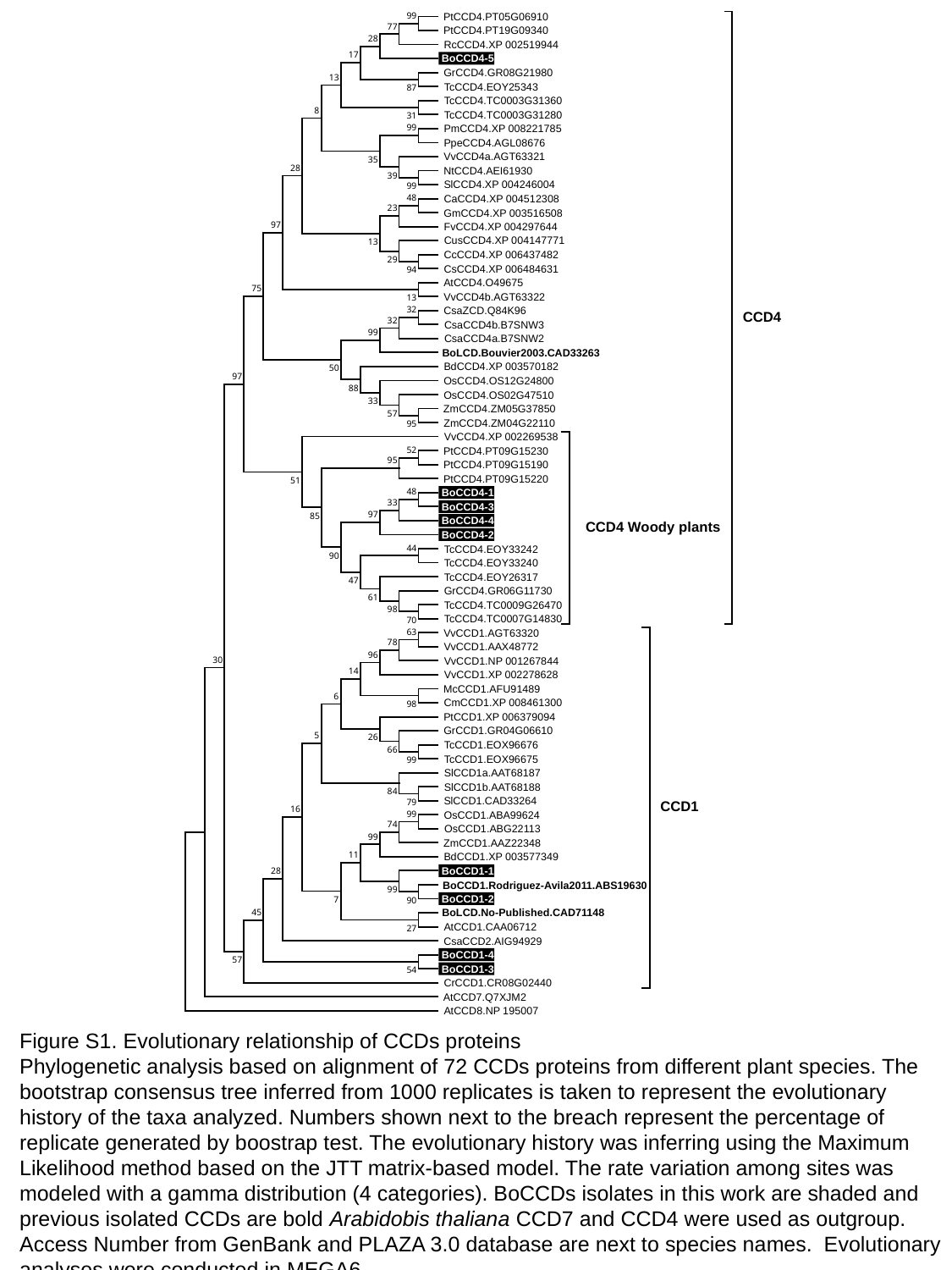

PtCCD4.PT05G06910
 PtCCD4.PT19G09340
 RcCCD4.XP 002519944
 BoCCD4-5
 GrCCD4.GR08G21980
 TcCCD4.EOY25343
 TcCCD4.TC0003G31360
 TcCCD4.TC0003G31280
 PmCCD4.XP 008221785
 PpeCCD4.AGL08676
 VvCCD4a.AGT63321
 NtCCD4.AEI61930
 SlCCD4.XP 004246004
 CaCCD4.XP 004512308
 GmCCD4.XP 003516508
 FvCCD4.XP 004297644
 CusCCD4.XP 004147771
 CcCCD4.XP 006437482
 CsCCD4.XP 006484631
 AtCCD4.O49675
 VvCCD4b.AGT63322
 CsaZCD.Q84K96
CCD4
 CsaCCD4b.B7SNW3
 CsaCCD4a.B7SNW2
 BoLCD.Bouvier2003.CAD33263
 BdCCD4.XP 003570182
 OsCCD4.OS12G24800
 OsCCD4.OS02G47510
 ZmCCD4.ZM05G37850
 ZmCCD4.ZM04G22110
 VvCCD4.XP 002269538
 PtCCD4.PT09G15230
 PtCCD4.PT09G15190
 PtCCD4.PT09G15220
 BoCCD4-1
 BoCCD4-3
 BoCCD4-4
CCD4 Woody plants
 BoCCD4-2
 TcCCD4.EOY33242
 TcCCD4.EOY33240
 TcCCD4.EOY26317
 GrCCD4.GR06G11730
 TcCCD4.TC0009G26470
 TcCCD4.TC0007G14830
 VvCCD1.AGT63320
 VvCCD1.AAX48772
 VvCCD1.NP 001267844
 VvCCD1.XP 002278628
 McCCD1.AFU91489
 CmCCD1.XP 008461300
 PtCCD1.XP 006379094
 GrCCD1.GR04G06610
 TcCCD1.EOX96676
 TcCCD1.EOX96675
 SlCCD1a.AAT68187
 SlCCD1b.AAT68188
 SlCCD1.CAD33264
 OsCCD1.ABA99624
 OsCCD1.ABG22113
 ZmCCD1.AAZ22348
 BdCCD1.XP 003577349
 BoCCD1-1
 BoCCD1.Rodriguez-Avila2011.ABS19630
 BoCCD1-2
 BoLCD.No-Published.CAD71148
 AtCCD1.CAA06712
99
77
28
17
13
87
8
31
99
35
28
39
99
48
23
97
13
29
94
75
13
32
32
99
50
97
88
33
57
95
52
95
51
48
33
97
85
44
90
47
61
98
70
63
78
96
30
14
6
98
5
26
66
99
84
79
CCD1
16
99
74
99
11
28
99
7
90
45
27
 CsaCCD2.AIG94929
 BoCCD1-4
57
 BoCCD1-3
54
 CrCCD1.CR08G02440
 AtCCD7.Q7XJM2
 AtCCD8.NP 195007
Figure S1. Evolutionary relationship of CCDs proteins
Phylogenetic analysis based on alignment of 72 CCDs proteins from different plant species. The bootstrap consensus tree inferred from 1000 replicates is taken to represent the evolutionary history of the taxa analyzed. Numbers shown next to the breach represent the percentage of replicate generated by boostrap test. The evolutionary history was inferring using the Maximum Likelihood method based on the JTT matrix-based model. The rate variation among sites was modeled with a gamma distribution (4 categories). BoCCDs isolates in this work are shaded and previous isolated CCDs are bold Arabidobis thaliana CCD7 and CCD4 were used as outgroup. Access Number from GenBank and PLAZA 3.0 database are next to species names.  Evolutionary analyses were conducted in MEGA6
