# Supplementary material for: De novo transcriptome sequencing in Bixa orellana to identify genes involved in methylerythritol phosphate, carotenoid and bixin biosynthesis
Source: BMC Genomics. 2015 Oct 28;16:877. doi: 10.1186/s12864-015-2065-4 (PMC4625570; doi:10.1186/s12864-015-2065-4)
Supplement: Additional file 2: Figure S1. — Evolutionary relationship of CCDs proteins. Figure S2. Evolutionary relationship of ALDH proteins. Figure S3. Evolutionary relationship of SABATH methyltransferases proteins. Figure S4. Evolutionary relationship of DXS proteins. (ZIP 410 kb) [file 12864_2015_2065_MOESM2_ESM.zip › Additional file 2_Figure S2.pptx]

## Slide 1
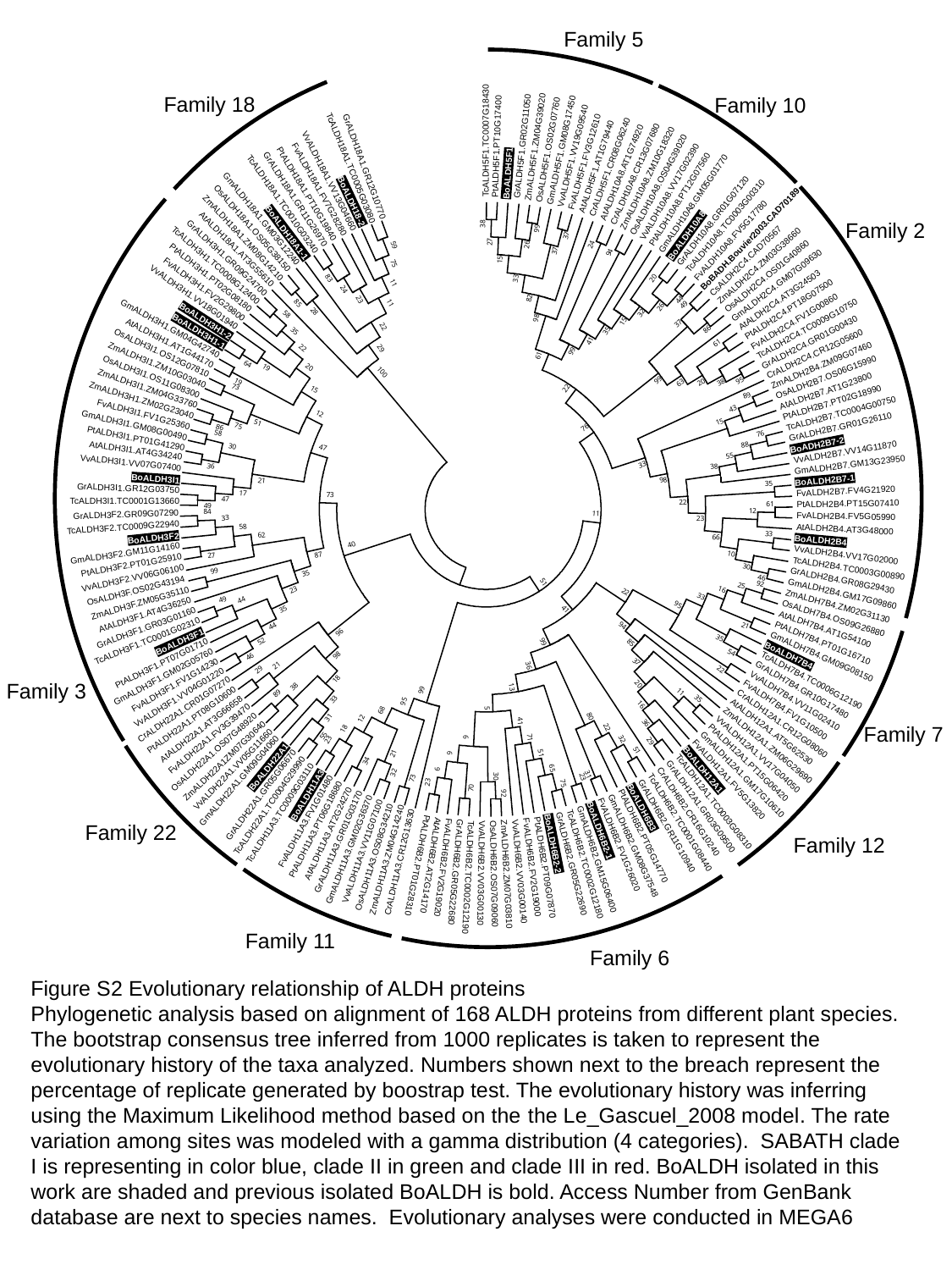

Family 5
TcALDH5F1.TC0007G18430
GrALDH5F1.GR02G11050
PtALDH5F1.PT10G17400
ZmALDH5F1.ZM04G39020
OsALDH5F1.OS02G07760
GmALDH5F1.GM08G17450
VvALDH5F1.VV19G09540
FvALDH5F1.FV3G12610
AtALDH5F1.AT1G79440
CrALDH5F1.CR08G06240
AtALDH10A8.AT1G74920
BoALDH5F1
CrALDH10A8.CR13G07680
ZmALDH10A8.ZM10G18320
OsALDH10A8.OS04G39020
VvALDH10A8.VV17G02390
PtALDH10A8.PT12G07560
GmALDH10A8.GM05G01770
GrALDH10A8.GR01G07120
38
TcALDH10A8.TC0003G00310
99
37
BoALDH10A8
BoBADH.Bouvier2003.CAD70189
FvALDH10A8.FV5G17780
27
26
24
37
94
15
CsALDH2C4.CAD70567
ZmALDH2C4.ZM03G38660
OsALDH2C4.OS01G40860
33
20
GmALDH2C4.GM07G09630
82
AtALDH2C4.AT3G24503
44
49
26
PtALDH2C4.PT18G07500
32
98
FvALDH2C4.FV1G00860
15
37
TcALDH2C4.TC0009G10750
86
35
41
GrALDH2C4.GR01G00430
61
99
CrALDH2C4.CR12G05600
61
ZmALDH2B4.ZM09G07460
OsALDH2B7.OS06G15990
99
95
63
20
38
22
AtALDH2B7.AT1G23800
89
GrALDH18A1.GR12G10770
TcALDH18A1.TC0005G03080
VvALDH18A1.VV13G04660
FvALDH18A1.FV7G28280
PtALDH18A1.PT10G19840
GrALDH18A1.GR11G26970
BoALDH18-2
TcALDH18A1.TC0010G03240
GmALDH18A1.GM03G12240
OsALDH18A1.OS05G38150
BoALDH18A1-1
ZmALDH18A1.ZM08G14210
59
AtALDH18A1.AT3G55610
GrALDH3H1.GR09G24700
75
TcALDH3H1.TC0008G12400
PtALDH3H1.PT02G08180
83
11
FvALDH3H1.FV2G29800
24
VvALDH3H1.VV18G01940
23
11
85
28
58
BoALDH3H1-2
22
GmALDH3H1.GM04G42740
BoALDH3H1-1
35
AtALDH3H1.AT1G44170
22
29
OsALDH3I1.OS12G07810
ZmALDH3I1.ZM10G03040
64
20
19
100
OsALDH3I1.OS11G08300
19
73
ZmALDH3I1.ZM04G33760
15
ZmALDH3H1.ZM02G23040
FvALDH3I1.FV1G25360
12
51
GmALDH3I1.GM08G00490
75
86
58
PtALDH3I1.PT01G41290
30
47
AtALDH3I1.AT4G34240
VvALDH3I1.VV07G07400
36
21
17
73
40
PtALDH2B7.PT02G18990
43
TcALDH2B7.TC0004G00750
15
GrALDH2B7.GR01G26110
76
76
BoADH2B7-2
88
VvALDH2B7.VV14G11870
55
GmALDH2B7.GM13G23950
33
38
BoALDH2B7-1
98
35
FvALDH2B7.FV4G21920
22
PtALDH2B4.PT15G07410
61
12
FvALDH2B4.FV5G05990
23
AtALDH2B4.AT3G48000
33
66
BoALDH2B4
10
VvALDH2B4.VV17G02000
30
TcALDH2B4.TC0003G00890
46
GrALDH2B4.GR08G29430
92
25
16
GmALDH2B4.GM17G09860
33
95
ZmALDH7B4.ZM02G31130
OsALDH7B4.OS09G26880
21
AtALDH7B4.AT1G54100
35
PtALDH7B4.PT01G16710
54
BoALDH7B4
GmALDH7B4.GM09G08150
22
TcALDH7B4.TC0006G12190
GrALDH7B4.GR10G17480
11
35
VvALDH7B4.VV11G02410
FvALDH7B4.FV1G10500
36
CrALDH12A1.CR12G08060
AtALDH12A1.AT5G62530
29
ZmALDH12A1.ZM06G29690
VvALDH12A1.VV17G04050
PtALDH12A1.PT15G06420
BoALDH12A1
GmALDH12A1.GM17G10610
FvALDH12A1.FV5G13820
TcALDH12A1.TC0003G08310
GrALDH12A1.GR03G09500
11
22
41
94
99
85
37
36
20
13
16
5
80
41
22
9
71
32
51
9
51
21
34
65
6
32
31
30
23
73
23
75
70
92
BoALDH6B3
CrALDH6B2.CR16G10240
FvALDH11A3.FV1G01480
TcALDH6B2.TC0001G08440
GrALDH6B2.GR11G10940
PtALDH11A3.PT06G18680
BoALDH6B2-1
AtALDH11A3.AT2G24270
PtALDH6B2.PT05G14770
GrALDH11A3.GR01G03170
BoALDH6B2-2
FvALDH6B2.FV1G26020
GmALDH6B2.GM08G37548
GmALDH11A3.GM02G36370
VvALDH11A3.VV11G07100
OsALDH11A3.OS08G34210
GmALDH6B2.GM15G06400
ZmALDH11A3.ZM04G14240
CrALDH11A3.CR12G13630
GrALDH6B2.GR05G22690
TcALDH6B2.TC0002G12180
AtALDH6B2.AT2G14170
PtALDH6B2.PT01G28310
FvALDH6B2.FV2G19000
PtALDH6B2.PT09G07870
FvALDH6B2.FV2G19020
VvALDH6B2.VV03G00140
GrALDH6B2.GR05G22680
VvALDH6B2.VV03G00130
OsALDH6B2.OS07G09060
ZmALDH6B2.ZM07G03810
TcALDH6B2.TC0002G12190
BoALDH3I1
GrALDH3I1.GR12G03750
47
TcALDH3I1.TC0001G13660
49
84
GrALDH3F2.GR09G07290
33
TcALDH3F2.TC0009G22940
58
62
BoALDH3F2
GmALDH3F2.GM11G14160
87
27
PtALDH3F2.PT01G25910
99
35
VvALDH3F2.VV06G06100
51
OsALDH3F.OS02G43194
23
49
44
ZmALDH3F.ZM05G35110
35
AtALDH3F1.AT4G36250
GrALDH3F1.GR03G01160
44
96
TcALDH3F1.TC0001G02310
BoALDH3F1
52
98
46
PtALDH3F1.PT07G01710
21
29
GmALDH3F1.GM02G05760
18
FvALDH3F1.FV1G14230
38
99
89
VvALDH3F1.VV04G01220
33
95
CrALDH22A1.CR01G07270
68
31
12
PtALDH22A1.PT08G10600
AtALDH22A1.AT3G66658
18
55
FvALDH22A1.FV3G39470
23
OsALDH22A1.OS07G48920
ZmALDH22A1ZM07G30640
BoALDH22A1
VvALDH22A1.VV05G11660
GmALDH22A1.GM09G04060
GrALDH22A1.GR05G06670
BoALDH11A3
TcALDH22A1.TC0004G29990
TcALDH11A3.TC0009G03110
Family 18
Family 10
Family 2
Family 3
Family 7
Family 22
Family 12
Family 11
Family 6
Figure S2 Evolutionary relationship of ALDH proteins
Phylogenetic analysis based on alignment of 168 ALDH proteins from different plant species. The bootstrap consensus tree inferred from 1000 replicates is taken to represent the evolutionary history of the taxa analyzed. Numbers shown next to the breach represent the percentage of replicate generated by boostrap test. The evolutionary history was inferring using the Maximum Likelihood method based on the the Le_Gascuel_2008 model. The rate variation among sites was modeled with a gamma distribution (4 categories). SABATH clade I is representing in color blue, clade II in green and clade III in red. BoALDH isolated in this work are shaded and previous isolated BoALDH is bold. Access Number from GenBank database are next to species names.  Evolutionary analyses were conducted in MEGA6
