# Supplementary material for: De novo transcriptome sequencing in Bixa orellana to identify genes involved in methylerythritol phosphate, carotenoid and bixin biosynthesis
Source: BMC Genomics. 2015 Oct 28;16:877. doi: 10.1186/s12864-015-2065-4 (PMC4625570; doi:10.1186/s12864-015-2065-4)
Supplement: Additional file 2: Figure S1. — Evolutionary relationship of CCDs proteins. Figure S2. Evolutionary relationship of ALDH proteins. Figure S3. Evolutionary relationship of SABATH methyltransferases proteins. Figure S4. Evolutionary relationship of DXS proteins. (ZIP 410 kb) [file 12864_2015_2065_MOESM2_ESM.zip › Additional file 2_Figure S3.pptx]

## Slide 1
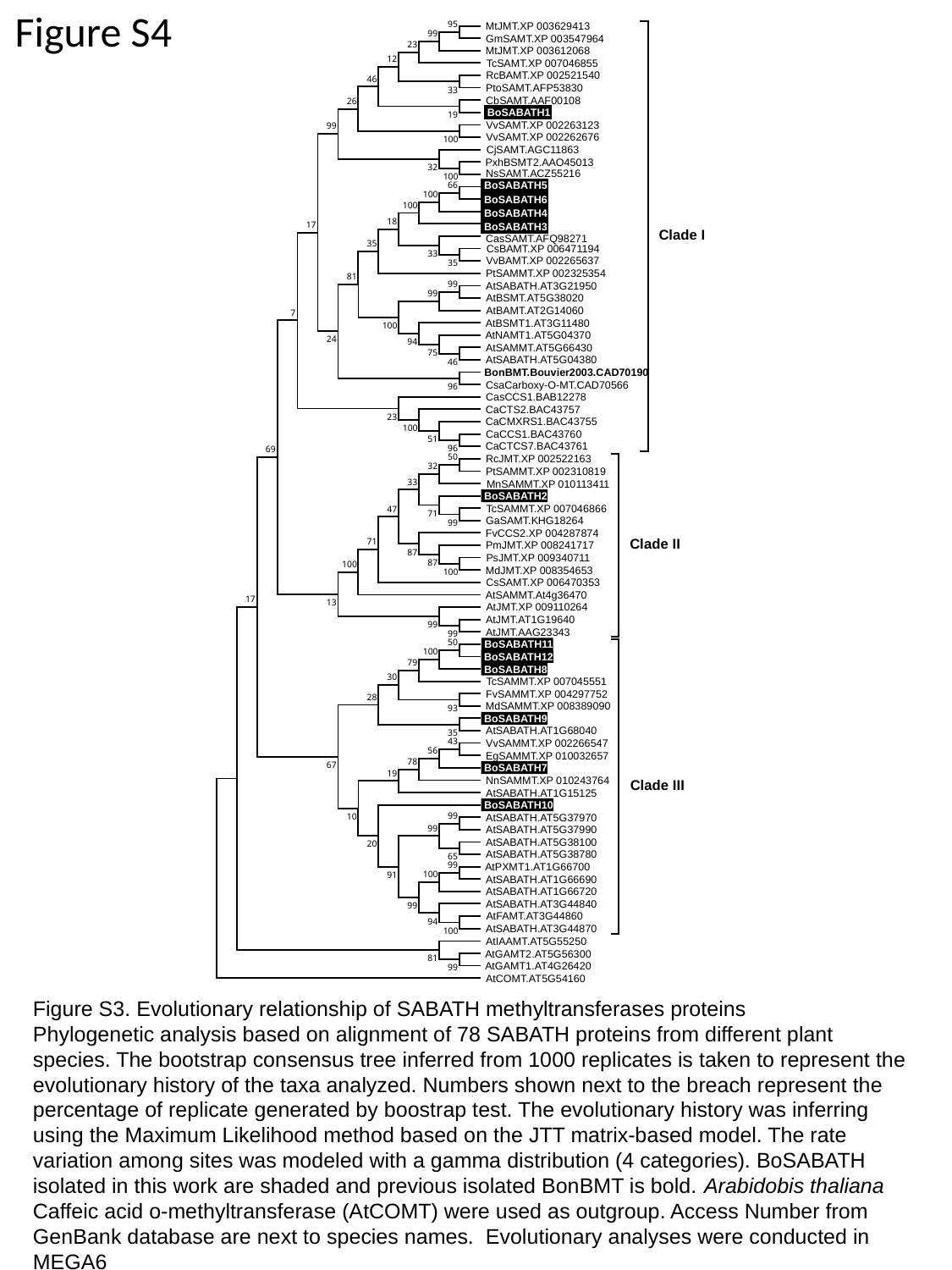

Figure S4
95
 MtJMT.XP 003629413
 GmSAMT.XP 003547964
 MtJMT.XP 003612068
 TcSAMT.XP 007046855
 RcBAMT.XP 002521540
 PtoSAMT.AFP53830
 CbSAMT.AAF00108
 BoSABATH1
 VvSAMT.XP 002263123
 VvSAMT.XP 002262676
 CjSAMT.AGC11863
 PxhBSMT2.AAO45013
 NsSAMT.ACZ55216
 BoSABATH5
 BoSABATH6
 BoSABATH4
 BoSABATH3
Clade I
 CasSAMT.AFQ98271
 CsBAMT.XP 006471194
 VvBAMT.XP 002265637
 PtSAMMT.XP 002325354
 AtSABATH.AT3G21950
 AtBSMT.AT5G38020
 AtBAMT.AT2G14060
 AtBSMT1.AT3G11480
 AtNAMT1.AT5G04370
 AtSAMMT.AT5G66430
 AtSABATH.AT5G04380
 BonBMT.Bouvier2003.CAD70190
 CsaCarboxy-O-MT.CAD70566
 CasCCS1.BAB12278
 CaCTS2.BAC43757
 CaCMXRS1.BAC43755
 CaCCS1.BAC43760
 CaCTCS7.BAC43761
 RcJMT.XP 002522163
 PtSAMMT.XP 002310819
 MnSAMMT.XP 010113411
 BoSABATH2
 TcSAMMT.XP 007046866
 GaSAMT.KHG18264
 FvCCS2.XP 004287874
Clade II
 PmJMT.XP 008241717
 PsJMT.XP 009340711
 MdJMT.XP 008354653
 CsSAMT.XP 006470353
 AtSAMMT.At4g36470
 AtJMT.XP 009110264
 AtJMT.AT1G19640
 AtJMT.AAG23343
 BoSABATH11
 BoSABATH12
 BoSABATH8
 TcSAMMT.XP 007045551
 FvSAMMT.XP 004297752
 MdSAMMT.XP 008389090
 BoSABATH9
 AtSABATH.AT1G68040
 VvSAMMT.XP 002266547
 EgSAMMT.XP 010032657
 BoSABATH7
 NnSAMMT.XP 010243764
 AtSABATH.AT1G15125
 BoSABATH10
 AtSABATH.AT5G37970
 AtSABATH.AT5G37990
 AtSABATH.AT5G38100
 AtSABATH.AT5G38780
99
23
12
46
33
26
19
99
100
32
100
66
100
100
18
17
35
33
35
81
99
99
7
100
24
94
75
46
96
23
100
51
96
69
50
32
33
47
71
99
71
87
87
100
100
17
13
99
99
50
100
79
30
28
93
35
43
56
78
67
19
Clade III
99
10
99
20
65
99
 AtPXMT1.AT1G66700
100
91
 AtSABATH.AT1G66690
 AtSABATH.AT1G66720
 AtSABATH.AT3G44840
99
 AtFAMT.AT3G44860
94
 AtSABATH.AT3G44870
100
 AtIAAMT.AT5G55250
 AtGAMT2.AT5G56300
81
 AtGAMT1.AT4G26420
99
 AtCOMT.AT5G54160
Figure S3. Evolutionary relationship of SABATH methyltransferases proteins
Phylogenetic analysis based on alignment of 78 SABATH proteins from different plant species. The bootstrap consensus tree inferred from 1000 replicates is taken to represent the evolutionary history of the taxa analyzed. Numbers shown next to the breach represent the percentage of replicate generated by boostrap test. The evolutionary history was inferring using the Maximum Likelihood method based on the JTT matrix-based model. The rate variation among sites was modeled with a gamma distribution (4 categories). BoSABATH isolated in this work are shaded and previous isolated BonBMT is bold. Arabidobis thaliana Caffeic acid o-methyltransferase (AtCOMT) were used as outgroup. Access Number from GenBank database are next to species names.  Evolutionary analyses were conducted in MEGA6
